# Coumarin Dimer Is an Effective Photomechanochemical AND Gate for Small-Molecule Release

**DOI:** 10.1021/jacs.3c07883

**Published:** 2023-10-11

**Authors:** Xiaojun He, Yancong Tian, Robert T. O’Neill, Yuanze Xu, Yangju Lin, Wengui Weng, Roman Boulatov

**Affiliations:** † Department of Chemistry, College of Chemistry and Engineering, Xiamen University, Xiamen, Fujian 361005, China; ‡ Department of Chemistry, University of Liverpool, Crown Street, Liverpool L69 7ZD, U.K.

## Abstract

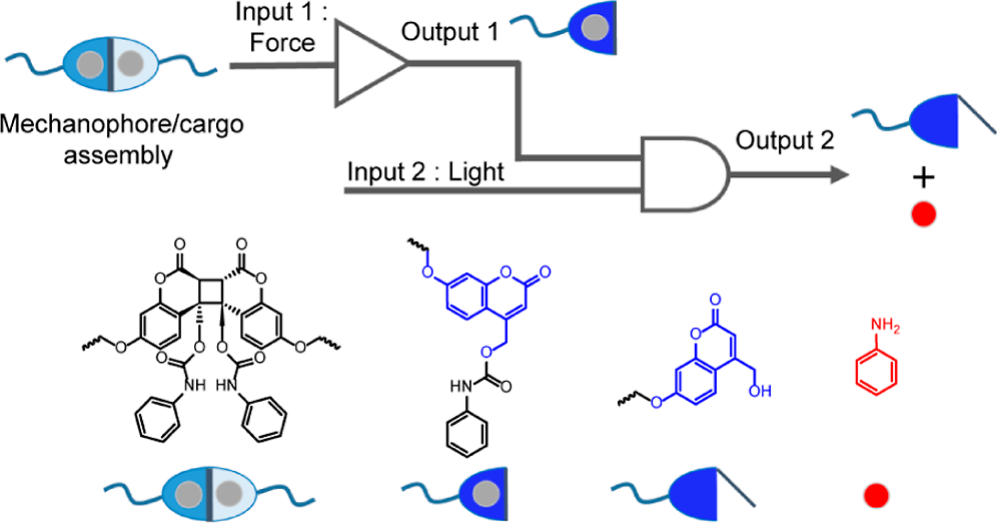

Stimulus-responsive gating of chemical reactions is of considerable practical and conceptual interest. For example, photocleavable protective groups and gating mechanophores allow the kinetics of purely thermally activated reactions to be controlled optically or by mechanical load by inducing the release of small-molecule reactants. Such release only in response to a sequential application of both stimuli (photomechanochemical gating) has not been demonstrated despite its unique expected benefits. Here, we describe computational and experimental evidence that coumarin dimers are highly promising moieties for realizing photomechanochemical control of small-molecule release. Such dimers are transparent and photochemically inert at wavelengths >300 nm but can be made to dissociate rapidly under tensile force. The resulting coumarins are mechanochemically and thermally stable, but rapidly release their payload upon irradiation. Our DFT calculations reveal that both strain-free and mechanochemical kinetics of dimer dissociation are highly tunable over an unusually broad range of rates by simple substitution. In head-to-head dimers, the phenyl groups act as molecular levers to allow systematic and predictable variation in the force sensitivity of the dissociation barriers by choice of the pulling axis. As a proof-of-concept, we synthesized and characterized the reactivity of one such dimer for photomechanochemically controlled release of aniline and its application for controlling bulk gelation.

## Introduction

**Figure 1:**
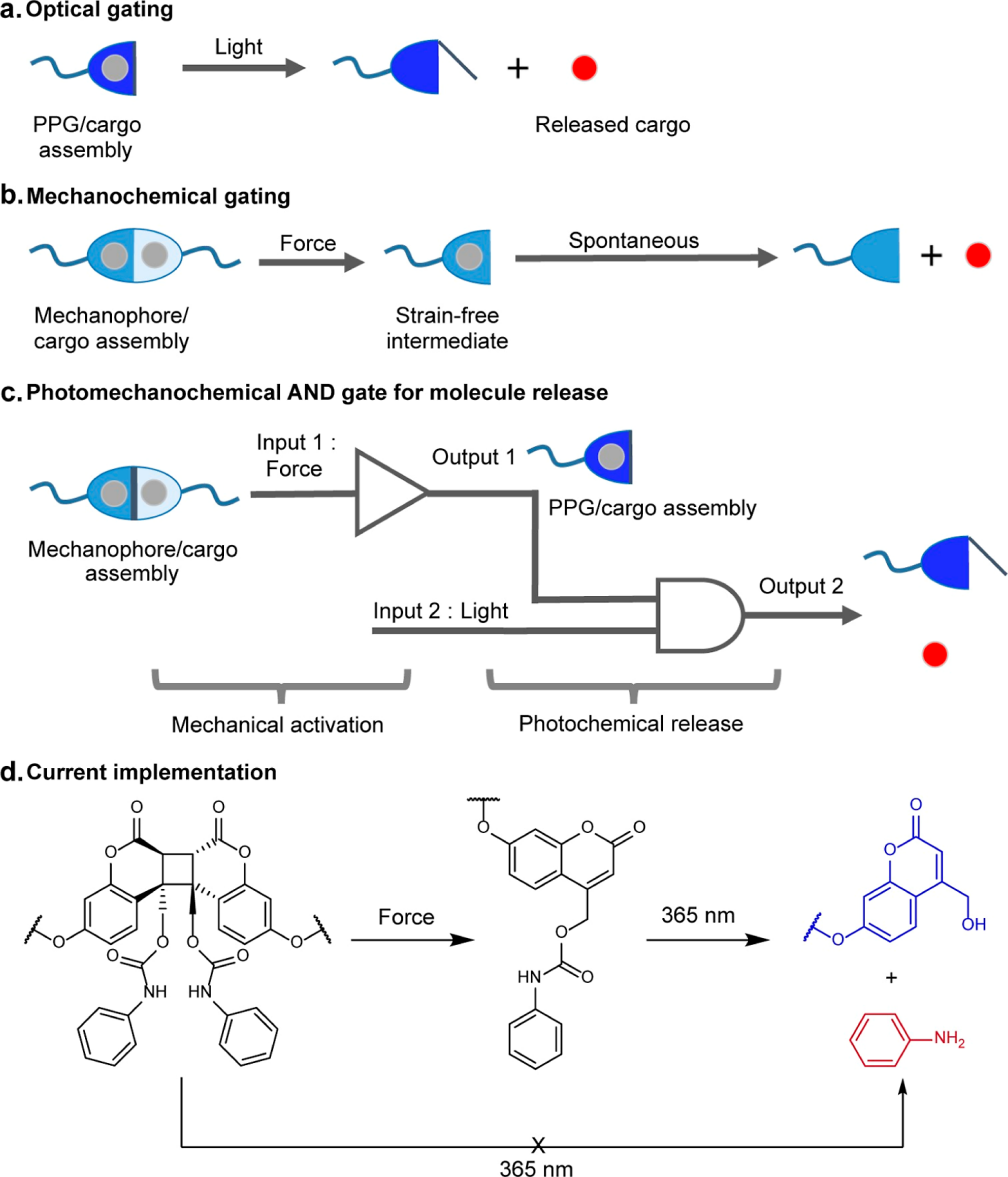
Schematic representation of gated molecular release and structural design of the photomechanochemical AND gate. (a) Optically gated release with PPGs. (b) Mechanically gated release. (c) Photomechanochemical AND gate. (d) Coumarin dimer for photomechanochemically gated aniline release: the dimer is photochemically stable at >300 nm but dissociates rapidly when pulled at the phenoxy oxygens; the intermediate coumarin is mechanochemically inert but releases aniline upon irradiation at 365 nm.

Gated or caged reactants are of considerable fundamental and practical interest. The best developed is photochemical gating, exemplified by photocleavable protective groups (PPGs, Figure 1a).^1^ PPGs release diverse organic molecules, inorganic ions, and simple gases (NO, CO, and H_2_S) upon light irradiation, which then can initiate downstream reactions with precise temporospatial control in diverse environments. Much less explored but potentially offering complementary capabilities is mechanochemical gating.^2−5^ It relies on the capacity of an externally generated stretching force to accelerate the dissociation or isomerization of diverse reactive moieties. In the most common implementation of mechanochemical gating, a rapid mechanochemical reaction of a moiety experiencing an above-threshold force yields a strain-free product that spontaneously releases a small molecule (Figure 1b).^6−9^ The development of mechanochemical gating is motivated by both practical goals, such as new polymeric materials capable of autonomic reporting or repair of mechanical damage^10^ and new drug-delivery systems;^8^ and by fundamental interest. The latter includes mapping the distribution of single-chain forces in mechanically loaded soft materials,^11^ the propagation of molecular strain across molecular networks,^12−14^ and understanding mechanochemical reaction networks^15,16^ and feedback loops.^17^

Mechanochemical gating of a photoisomerization reaction^18,19^ and optical gating of mechanochemical dissociations are known.^20,21^ Conversely, attempts to control the kinetics of small-molecule release by a combination of optical and mechanical stimuli have not been reported despite such a combination offering important advantages over single-stimulus implementations. Compared to a mechanochemically gated reaction, photomechanochemical gating overcomes the presently limited control over localization of the reaction-triggering threshold force in bulk materials. For example, despite considerable recent progress, medium-frequency ultrasound, which is currently the most controlled method of applying stretching force to macromolecules in solution, is limited to spatial resolution of >0.1 mm and temporal resolution of ∼1 s.^22^ Likewise, photomechanochemical gating potentially overcomes the seemingly contradictory set of requirements for an optimal PPG:^1^ groups cleavable with longer-wavelength light are advantageous due to the usually longer penetration depth of such light in both biological tissue and many polymeric materials. This has to be balanced against the reduced stability of PPGs activatable with wavelengths >500 nm under ambient light or the technical complexity of two-photon activation. Finally, photomechanochemical gating allows actions that are currently either impossible, such as temporospatial control over initiating cross-linking within specific subvolumes of a polymeric material experiencing load above a predefined threshold,^23^ or that are impractically complex, such as mechanochemical soft lithography.^24,25^

Longer-term exploitation of the potential of photomechanochemical reaction gating requires the design and characterization of photochemically inert moieties whose reactions are accelerated by a stretching force and yield a chromophore capable of initiating further thermally activated reactions upon light irradiation (Figure 1c). The contemporary interest in both optically and mechanically controlled delivery of small-molecule payloads makes the design and characterization of organic mechanochromic reactions that yield PPGs a particularly advantageous starting point.

Here, we describe experimental and computational evidence suggesting that the coumarin dimers are highly promising moieties for realizing photomechanochemical control of small-molecule release. Such dimers are transparent and photochemically inert at wavelengths >300 nm but can be made to dissociate rapidly under tensile force of varying thresholds. The resulting coumarins are mechanochemically and thermally stable, but rapidly release their payload upon irradiation. In other words, a coumarin dimer is a photomechanochemical AND gate responsive to only a sequential application of tensile load and irradiation with light (Figure 1c,d).

In the rest of this paper, we first describe the detailed quantum-chemical characterization of the mechanochemical dissociation mechanisms and structure–reactivity relationships in diverse coumarin dimers that suggest that such dimers may be useful in the broader range of loading scenarios than any other force-responsive molecular moieties identified to date. We then demonstrate the application of one such dimer for the photomechanochemically controlled release of a small molecule (aniline) and its use for triggering bulk gelation. This report focuses primarily on the mechanochemical component of our gate because photochemical fragmentation of (coumarin-4-yl)methyl groups has been studied extensively^1^ and is not affected by incorporation into a polymer chain.^26^

## Results and Discussion

### Mechanochemical Mechanisms and Structure/Reactivity Relationships in Coumarin Dimers from DFT Calculations

A coumarin dimer may have a broader utility in polymer mechanochemistry than any known alternative. Its dissociation can be effected both photo-^27^ and mechanochemically,^28^ and the large differences in the absorption and emission spectra of the dimer and the monomer enable strong photo-^29^ and mechanochromism and load-induced fluorescence.^30^ In addition to being a photomasking group, coumarins photodimerize readily, thus allowing optical healing of mechanically degraded material.^26^ The capacity to tune the optical properties of coumarin by peripheral substitution is highly developed,^29,31^ as is its synthetic chemistry.^32−36^ Against this potential, the puzzlingly limited use of the coumarin dimer in polymer mechanochemistry to date^28,30,37^ may be attributed to the lack of data relating the structure of the dimer to its thermal and mechanochemical stability.

**Figure 2:**
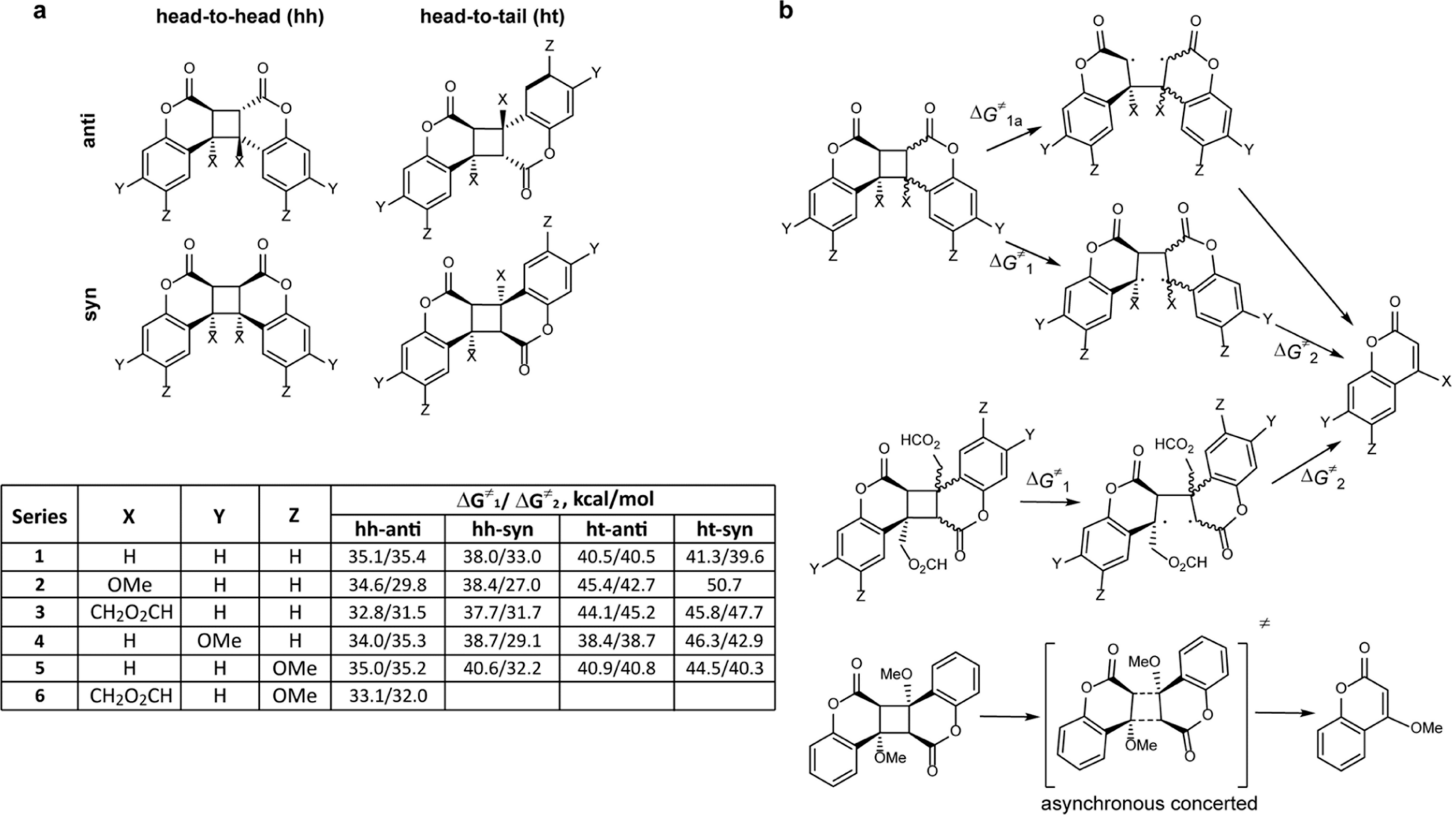
Summary of the results of DFT calculations of coumarin-dimer dissociation kinetics. (a) Isomers of the dimers studied, and the free energies of the dissociation transition states relative to each dimer. (b) Computed dissociation mechanisms. All results are at (u)MPW1K/6-31+G(d) in the gas phase. Cartesian coordinates of the minimum-energy conformers of all kinetically significant stationary states are tabulated in suppdata.mat (Supporting Information).

To facilitate the adoption of coumarin dimers in mechanochemical research^11,12^ and technology,^38^ we calculated the dissociation mechanisms and activation free energies of the four known isomers of the parent coumarin dimer and six of its derivative (Figure 2), as a function of stretching force along different pulling axes. The sensitivity of some mechanochemical reactions to which pair of atoms of the reacting moiety the force is applied to is well established.^39−45^ Conversely, the molecular structural basis of this sensitivity or the contributions of competing reaction mechanisms to it are little understood.^12^

We are aware of no literature reports of measured activation free energies of dissociation, Δ*G*_d_^⧧^, of any coumarin dimer, which precludes benchmarking the capacity of different functionals to reproduce experimental kinetics. We previously demonstrated that calculations at (u)MPW1K/6-31+G(d) level of DFT in the gas phase correctly reproduced the measured mechanochemical kinetics of dissociation of cinnamate dimers^46^ and dicarboxy bicyclooctanes,^5^ both of which include stepwise scission of the cyclobutane core, as well as the mechanism and activation energies of dissociation of dimethyl *trans*-1,2-dicarboxycyclobutane calculated at the (u)CCSD/aug-pVTZ level.^5^ Consequently, we used (u)MPW1K/6-31+G(d) for all calculations in this work. The minimum-energy dissociation mechanisms of all four isomers of the unsubstituted coumarin dimer calculated with CAM-B3LYP, MPW1KCIS1, and BMK functionals and the 6-31+G(d) basis set in vacuum were identical to those with MPW1K/6-31+G(d), and the activation free energies of dissociation with MPW1K, CAM-B3LYP, and MPW1KCIS1 were nearly identical and uniformly lower than those with BMK (Table S1).

### Force-Free Reactions

In the absence of external force, every studied dimer but one dissociate in two steps (Figure 2), traversing a pair of biradical transition states separated by a shallow energy minimum (<5 kcal/mol relative to the most stable TS). In head-to-head (hh) dimers, the free energy of the transition state for dissociation of the distal C–C bond (Δ*G*_1a_^⧧^, Figure 2b) is 10.8–16.3 kcal/mol higher than that for the dissociation of the proximal C–C bond in the dimer (Δ*G*_1_^⧧^) or the remaining scissile C–C bond in the corresponding intermediate (Δ*G*_2_^⧧^). The transition states for asynchronous single-step dissociation of head-to-tail dimers of the unsubstituted coumarin are 13.3 and 16.1 kcal/mol above the least-stable stepwise TSs, making concerted dissociations kinetically insignificant. Our systematic search for the stepwise dissociation mechanism of the *syn* head-to-tail dimer of 4-methoxy coumarin, ht-*syn*-**2**, failed to locate such a path, suggesting that this dimer is unique in dissociating only by an asynchronous concerted mechanism (bottom reaction, Figure 2b), which probably explains its exceptional inertness at all forces (see below).

Dissociation of all dimers requires activation energies >32.8 kcal/mol, suggesting that all would withstand conventional polymer melt processing without dissociation.^47^ As such, the dimers are likely better suited for yielding commercial mechanochromic polymers than the currently popular Diels–Alder adducts.^15,48,49^ Across all substituents studied, hh-*anti* dimers are the most dissociatively labile, and ht-*syn* analogues are the least dissociatively labile, with substituents increasing this difference from 6.7 kcal/mol in parent dimers to 17.9 kcal/mol in the dimers of 4-methylformate coumarin, series **3** (Figure 2a). For each relative orientation of the two coumarin moieties (hh vs ht), substitution affects Δ*G*_d_^⧧^ moderately, with the activation energies varying within the 4.5–6.7 kcal/mol range across the studied series.

### Force-Dependent Activation Free Energies

**Figure 3:**
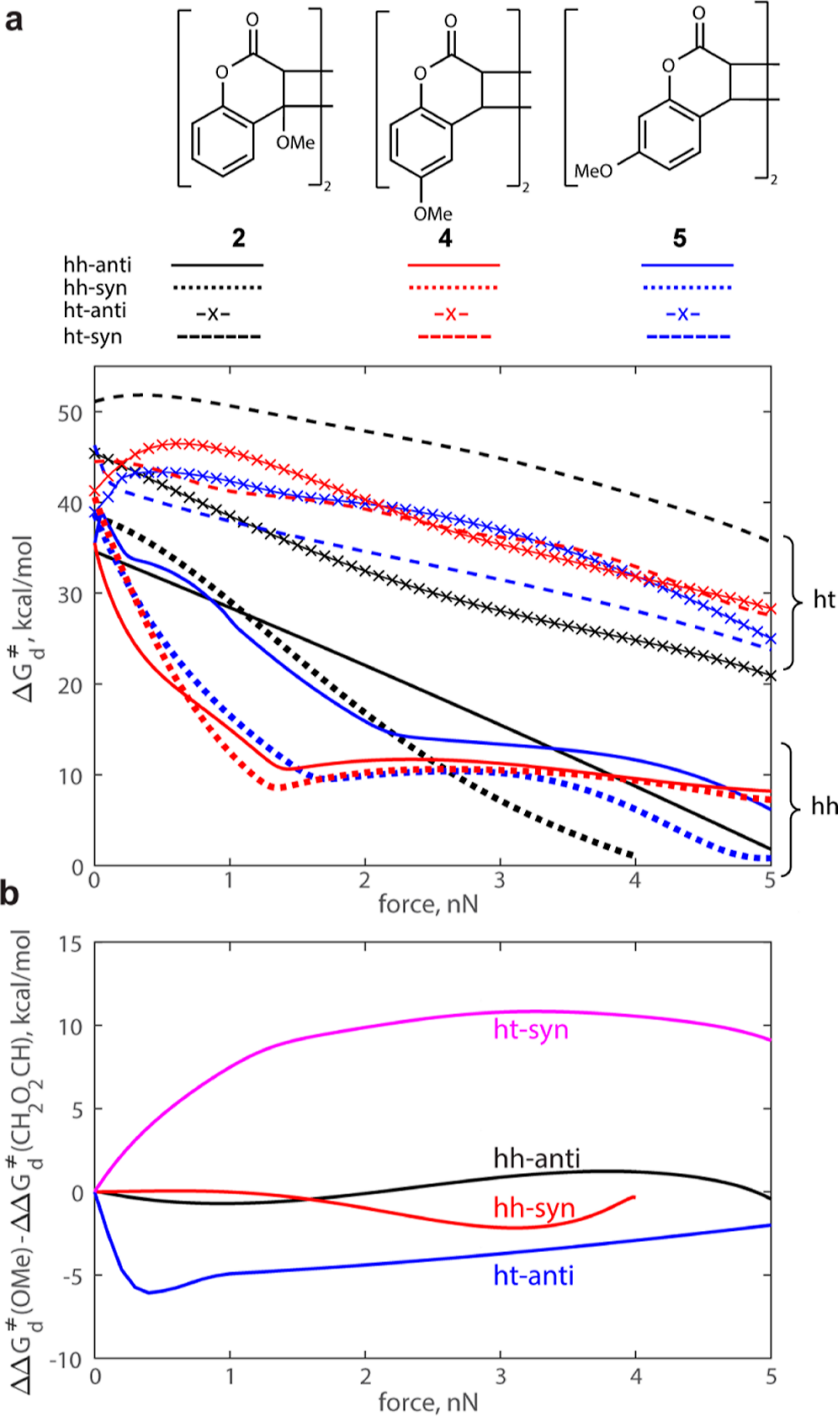
Summary of calculated structure–mechanochemical reactivity relationships in coumarin dimers. (a) Force-dependent activation free energies of dimer dissociation, Δ*G*_d_^⧧^, for series **2**, **4**, and **5**. Dissociation of ht-*syn*-**2**, ht-*anti*-**4**, and ht-*anti*-**5** is inhibited by tensile force <0.38, <0.1, and <0.46 nN, respectively, because below these thresholds the formation of the rate-determining transition states requires contraction of the ensemble-average _MeO_C···C_OMe_ distance across which the force acts. This contraction reflects the relatively high abundance of thermally accessible conformers with short _MeO_C···C_OMe_ distances in these TSs. Forces above the thresholds eliminate such short conformers,^54,55^ elongating the corresponding ensemble-average _MeO_C···C_OMe_ distances beyond those in the reactants. The resulting force-dependent stabilization of these TSs relative to the reactants accelerates dissociation. Nonmonotonically force-dependent Δ*G*^⧧^ were previously reported for dissociations of cinnamate dimers^46^ and a Diels–Alder adduct,^56^ isomerization of cyclobutenes,^57,58^ and several bimolecular mechanochemical reactions.^52,56,59^ (b) Effect of the composition of the force-transmitting substituents of cyclobutane (OMe vs CH_2_O_2_CH) on the extent of force-induced barrier lowering, ΔΔ*G*_d_^⧧^, as a function of applied force. Note that force <0.3 nN inhibits dissociation of ht-*syn*-**3** by the same mechanism as in ht-*syn*-**2**. For dissociations traversing the two TSs at least 3 kcal/mol apart, Δ*G*_d_^⧧^ equals the energy of the least-stable TS; for nearly isoenergetic TSs, Δ*G*_d_^⧧^ was calculated from those of individual barriers (Δ*G*_1_^⧧^ and Δ*G*_2_^⧧^, Figure 2b) by eq S1.^56^ The graphed data is tabulated in suppdata.mat (Supporting Information).

We calculated the force-dependent dissociation mechanisms and activation free energies for three pulling axes, defined by the terminal C atoms of the OMe or CH_2_O_2_CH substituents at the cyclobutane core (series **2** and **3**, Figure 2a); or one of the two distal positions on the phenyl rings (series **4**–**6**). The head-to-head vs head-to-tail orientation of the coumarin cores is the primary determinant of the sensitivity of Δ*G*_d_^⧧^ vs force for all pulling axes studied. Δ*G*_d_^⧧^ of all ht dimers (Figure 3a) are ∼4 times less sensitive to stretching force than those of the hh congeners, with the respective Δ*G*_d_^⧧^/force slopes averaging –(3.9 ± 0.9) kcal/mol/nN (0.27 ± 0.06 Å) vs –(13.0 ± 0.4) kcal/mol/nN (0.91 ± 0.02 Å), respectively (for hh isomers of series **4** and **5** dimers, the slopes are at force ≤1.5 nN only due to their highly nonmonotonic Δ*G*_d_^⧧^ vs force correlations, see below). These slopes correspond to a 2-fold acceleration of dissociation of ht dimers vs a 9-fold acceleration of hh dimers per 0.1 nN of applied force at 298 K.

The effect of the pulling axis on Δ*G*_d_^⧧^(f) of hh dimers can be attributed to phenyl rings acting as levers^50^ that amplify the structural differences between the reactant and TS1. For example, the average slopes of Δ*G*_d_^⧧^(f) for hh-*anti* dimers pulled at the cyclobutane (series **2** and **3**), C3,C10-phenyl (series **4**), and C2,C11-phenyl (series **5**) substituents are 0.46, 0.84, and 1.0 Å, respectively. These slopes correlate well with the elongation of the respective intermolecular distances from the strain-free reactant to TS1 of the same dimers (0.35, 0.67, and 1.05 Å). Such correlations enable the kinetic stabilities of each regioisomer in the absence of force, which are comparatively easy to obtain, to be extrapolated with useful accuracy over a broad range of applied forces. These extrapolations can guide the selection of the regioisomer whose mechanochemical stability best matches the desired loading scenario, including the range of forces needed to achieve the desired reaction rate, without the need for detailed and resource-intensive calculations of force-dependent kinetics.^51^

Conversely, in strain-free ht dimers, the _MeO_C···C_OMe_ coordinate of phenyl-bound OMe groups is effectively orthogonal to the scissile bond. Consequently, the less-force-sensitive dissociation barriers of ht dimers are dominated by a combination of differential distortions of the reactant and transition state geometries by force, and the reduction in the number of thermally accessible conformers.^17,48,52^ Such second-order effects are impossible to estimate from strain-free geometries.^53^

The remarkable uniformity of Δ*G*_d_^⧧^ vs force slopes in dimers covering a broad range of strain-free Δ*G*_d_^⧧^ (e.g., 0.27 ± 0.06 Å vs 38.7–50.7 kcal/mol for ht dimers) suggests that the pulling axis can control mechanochemical kinetics independently of the variation of the electronic structure of the derivatives caused by their distinct substitution pattern and reflected in the range in strain-free Δ*G*_d_^⧧^.

In contrast to several other mechanochemical reactions,^58^ the structure of the handles through which the extrinsic force is transmitted to the cyclobutane core affects the ΔΔ*G*_d_^⧧^(f) dependences only marginally (Figure 3b). The large difference in Δ*G*_d_^⧧^(f) of ht-*syn* dimers with OMe vs CH_2_OC(O)H handles (magenta line, Figure 3b) reflects the different dissociation mechanisms (concerted vs stepwise, respectively) rather than distribution of applied load across the molecular degrees of freedom of the handles, which explains such differences in other reactions.^56^

### Force-Dependent Dissociation Mechanisms

**Figure 4:**
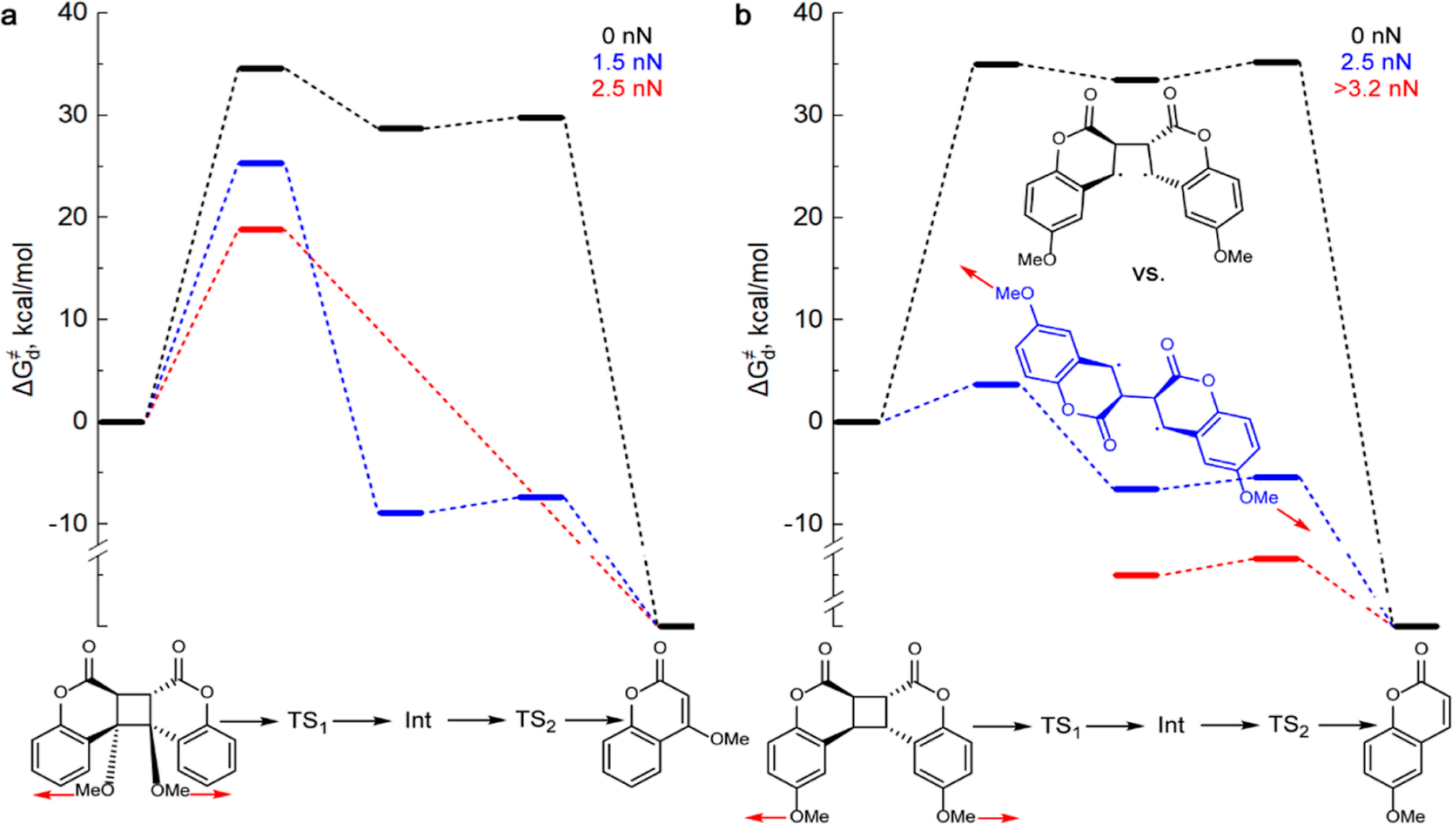
Force-induced changes in the dissociation mechanisms. (a) In dimers pulled at the cyclobutane substituents, the typically stronger stabilization by force of the outer dissociation barrier(s)^60^ eventually eliminates them to produce an asynchronous concerted scission above a threshold force (Table S2). (b) In dimers pulled at the phenyl substituents, the scission of the first cyclobutane bond above a threshold force is accompanied by rotation around the remaining scissile bond to produce Int and TS_2_ conformers (blue structure) with considerably longer _MeO_C···C_OMe_ distance than the dimer or TS_1_. This “extra” elongation greatly stabilizes Int and TS_2_ relative to the dimer, eventually eliminating the 1st barrier and the energy well of the intact dimer at *f*_max_.^55^ The long conformers of Int and TS_2_ exist in the absence of force but are 5–7 kcal/mol less stable than the compact conformer (black structure) and are kinetically insignificant below a threshold force. Note that the energy of Int and TS_2_ above *f*_max_ (3.2 nN in the illustrated example, red scheme) cannot be quantified relative to the dimer because the dimer does not exist at such forces. The energy of coumarins relative to the dimer coupled to an infinitely compliant stretching potential is similarly undefined.^55,73^ In all panels, red arrows indicate the extrinsic stretching force. The underlying data are tabulated in suppdata.mat (Supporting Information).

The sharp reduction in the slope of Δ*G*_d_^⧧^ vs force in hh dimers pulled at the phenyl substituents (blue and red solid and dotted lines in Figure 3a) reflects the change in the rate-determining step, as a consequence of the different force-sensitivities of sequential TSs in multibarrier dissociation mechanisms.^56,59^ The latter also causes the mechanism to change from stepwise to concerted as the force increases (Figure 4). Outer barriers of multibarrier dissociation reactions are usually more sensitive to force than inner barriers^60^ and disappear above a threshold force: series **2** and **3** dimers follow this pattern (Figure 4a).

Conversely, in series **4**–**6**, facile rotation around the remaining scissile bond in the intermediate means that above a threshold force (1.4–1.6 nN, depending on the isomer) the intermediate is more stable than the dimer^57^ and, the height of the second barrier is determined by the nearly force-independent structural difference between the intermediate and TS2 (Figure 4b). As the applied force increases further, the first barrier (and hence the dimer) disappears. This force-induced elimination of barriers contrasts with calculated mechanochemical dissociations of Diels–Alder adducts, which change from single-step concerted to multistep diradical as force increases.^56,61^ Because radical intermediates can be intercepted by adventitious radicals,^62−64^ mechanochemical reactions that avoid such intermediates are preferable for practical applications.^16^

### Dimers with Potentially Exploitable Mechanochemical Kinetics

**Figure 5:**
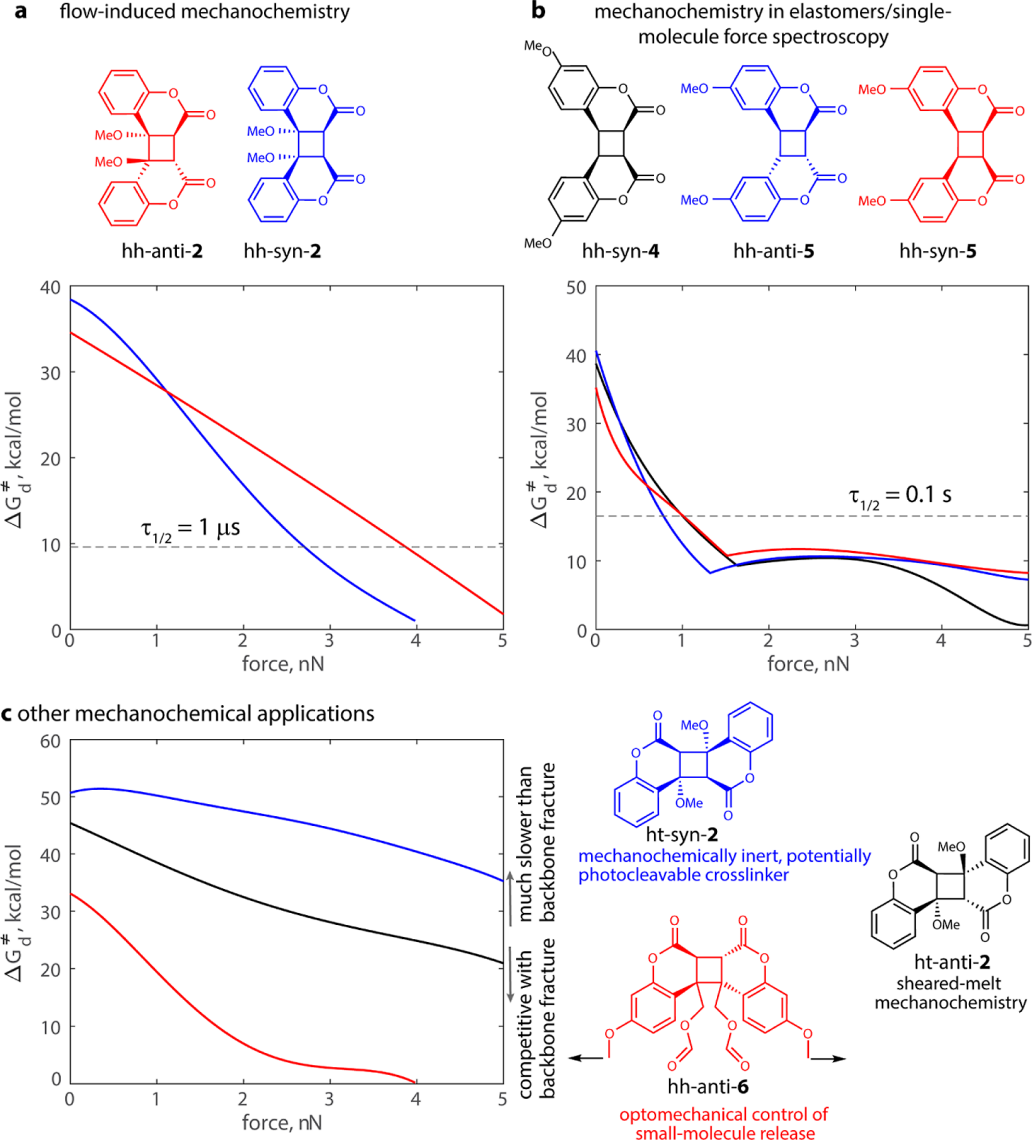
Coumarin-dimer derivatives with potentially exploitable mechanochemical kinetics. (a) In sonicated solutions, in solutions flowing through abrupt contractions, and in similar scenarios, chains remain stretched for sub μs periods, and only mechanochemical reactions with half-lives below ∼1 μs are observable. Monotonically decreasing Δ*G*_d_^⧧^ makes hh-*anti*-**2** and hh-*syn*-**2** the best coumarin dimers for quantifying the conditions responsible for such flow-induced mechanochemistry.^66^ (b) Corresponding time scales in mechanically loaded elastomers and in single-molecule force spectroscopy are on the order of 100 ms, but the accessible forces are lower than in flow-induced mechanochemistry.^12−14^ (c) At > 1 nN, Δ*G*_d_^⧧^ of ht-*syn*-**2** remains above that of mechanochemical homolysis of common backbone bonds (C–C, C–O, and C–N, average estimated Δ*G*_d_^⧧^ ∼ 24 kcal/mol at 5 nN),^16,46,66^ making it the only dimer studied that is functionally inert to mechanochemical dissociation at any practically relevant force. In each panel, the colors of the Δ*G*_d_^⧧^ curves match those of the accompanying chemical structures.

The remarkable diversity of patterns of Δ*G*_d_^⧧^ vs force correlations across a series of closely related reactive moieties is unmatched by any other known mechanochrome. The data here allows systematic identification of the dimer best suited for generating quantifiable mechanochromic response under diverse practically relevant loading conditions.^65^ For example, application of force to vicinal substituents of the cyclobutane core of hh dimers (e.g., hh-*anti*-**2/3**) yields Δ*G*_d_^⧧^ that decreases monotonically to 0 (Figure 5a), making them ideal for quantitation of large (3–5 nN) highly transient (<1 μs) forces experienced by macromolecular solutes in solvent flows generated by cavitation^66^ or abrupt flow obstructions.^67−69^ The steeper dependence of Δ*G*_d_^⧧^ on the force at <2 nN achieved by pulling at distal phenyl substituents of hh dimers (series **4**–**6**, Figure 5b) makes them well suited for quantifying the lower single-chain forces estimated to persist on ms timescales in mechanically loaded elastomers.^12,14^ The best candidate for generating mechanochromic response in sheared polymer melts,^16^ such as those in melt processing, is ht-*anti*-**2** (black line, Figure 5c) because of its combination of high thermal stability (force-free Δ*G*_d_^⧧^ = 45.4 kcal/mol) and usefully force-sensitive Δ*G*_d_^⧧^ (5 kcal/mol/nN = 0.35 Å).

Note that the minimum force at which the dissociation activation energy, Δ*G*_d_^⧧^, decreases below these key thresholds does not correlate with the maximum applied force, *f*_max_, to which the energy minimum corresponding to the intact (if highly strained) dimer survives. The former determines if the dimer dissociation is observable under specific loading conditions, e.g., in sonicated solutions (<10 kcal/mol, *t*_1/2_ < 1 μs),^66^ in sheared melts (<14 kcal/mol, *t*_1/2_ < 1 ms at 300 K),^16^ or in single-molecule force experiments and loaded elastomers (<16.5 kcal/mol, *t*_1/2_ < 0.1 s).^13,15^ For example, hh-*syn*-**4** and hh-*anti*-**5** exist up to nearly identical *f*_max_ (3.1 and 3.2 nN, Table S2), yet hh-*syn*-**4** almost certainly dissociates in sonicated solutions (Δ*G*_d_^⧧^ < 10 kcal/mol at >1 nN), whereas hh-*anti*-**5** is likely stable in sonicated solutions or sheared melts at 300 K (Δ*G*_d_^⧧^ > 10 kcal/mol at >4.7 nN). Similarly, hh-*anti*-**4** and hh-*syn*-**4** have nearly identical Δ*G*_d_^⧧^ across 0–5.5 nN, yet their *f*_max_ differs by 1.5 nN (4.6 vs 3.1 nN). Our data adds to the growing body of evidence^70,71^ that mechanochemical kinetics cannot be approximated, even qualitatively, by the response of only the reactant state to the applied force. This makes the CoGEF method, which is the most common approach of using *f*_max_ to predict if a molecule reacts under force, not only conceptually suspect but also empirically misleading.

Δ*G*_d_^⧧^ of ht-*syn*-**2** (Figure 5c, blue) remains too high even at 5 nN to compete with dissociation of other backbone bonds,^16,66^ making this moiety a potentially photocleavable^72^ (with 254 nm light) cross-linker with mechanochemical stability that exceeds that of common backbone bonds at any force >1 nN.^16,66^

Applications of coumarin dimers for photomechanochemical control of small-molecule release restrict the composition and placement of the substituents bearing the releasable payload to the hydroxymethyl moiety at the cyclobutane core. This leaves the distal phenyl sites for connections to the polymer backbone. Of several plausible candidates, derivatives of head-to-head *anti* dimer **6** are readily accessible synthetically and their photochemistry has been studied extensively. Because force-dependence of Δ*G*_d_^⧧^ of hh-*anti*-**6** (red, Figure 5c) makes its dissociation mechanochemically competent both in sonicated solutions (Δ*G*_d_^⧧^ < 10 kcal/mol at >2 nN) and in bulk elastomers (the slope of Δ*G*_d_^⧧^ vs force >5 kcal/mol/nN at 0–3 nN), we chose this derivative for experimental demonstration of photomechanochemical control of small-molecule release.

### Experimental Demonstration of Photomechanochemically Controlled Release of Aniline

We tested experimentally the concept of photomechanochemical gating on polymer **P8** (*M*_n_ = 181.5 kDa, *D̵*_M_ = 1.71) and for comparison on the product of mechanochemical dissociation of **P8** at the coumarin dimer, **P7** (*M*_n_ = 136.2 kDa, *D̵*_M_ = 1.54, Figure 6a). We synthesized **P7** and **P8** by living radical polymerization of oligo(ethylene glycol) methyl methacrylate (*M*_n_ = 300 g/mol) with Cu/Me_6_TREN in dry DMSO from precursors **7** (Figures 6a and S1–S26) and **8** (dimer of **7**), respectively. See Figures S27–S30 for detailed characterization of the polymers.

**Figure 6:**
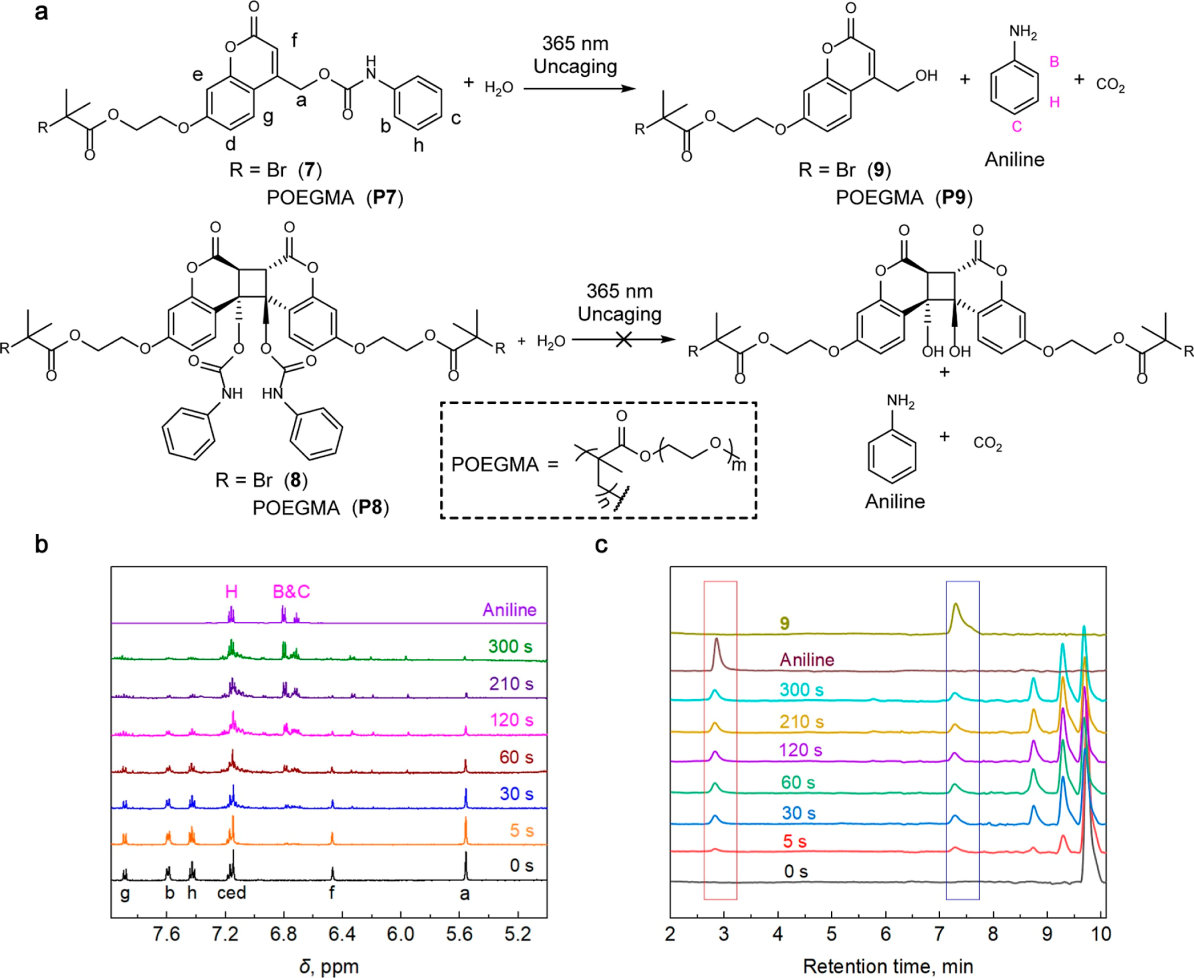
Photochemical release of aniline from coumarin **7**, its polymer **P7**, and their dimers **8** and **P8**. (a) Chemical reaction showing the assignment of the ^1^H NMR peaks (for **7** only). (b) ^1^H NMR spectra of a 0.4 mM solution of **7** in DMF-*d*_7_/D_2_O (2:1 by volume) at increasing irradiation (365 nm) times. For reference, the spectrum of a 1.5 mM solution of aniline in the same solvent is shown at the top. (c) 280 nm output of the UV–vis detector of HPLC of a 10 μM solution of **7** in 50% aqueous CH_3_OH at increasing irradiation (365 nm) times. For reference, chromatograms of a 2 μM solution of **9** in 50% aqueous CH_3_OH and a 5 μM solution of aniline in deionized water are shown as the top traces. The peaks at 8.8 and 9.3 min are homodimer **9**_2_ and a mixture of the mixed dimer of **7** and **9** and homodimer, **7**_2_, respectively.

We first established that the polymer backbone impacts negligibly the capacity of coumarin to act as a PPG by comparing the kinetics of aniline release from **P7** (Figures S31–S37) and its small-molecule precursor **7** (Figures 6 and S38 and S39) upon irradiation of their solutions at 365 nm. We characterized irradiated solutions of **7** by ^1^H NMR, absorption and emission spectroscopies, and HPLC (see the Supporting Information for a detailed description of the procedure, including calibration of HPLC for quantitation of aniline yield), and **P7** by absorption/emission spectroscopies and GPC. Our results are consistent with previous suggestions^1,26,74^ that release of the protected group competes with photodimerization of coumarin (Figure S36–S41), as illustrated, for example, by the appearance of HPLC peaks of the dimers in irradiated solutions of **9** and the shift of the molecular mass distribution of the irradiated solution of **P7** to higher mass (Figure S41). The resulting dimer is inert under irradiation conditions, thereby reducing the fraction of caged aniline that is released. At concentrations used in our experiments, 15–20% of coumarin/aniline adducts (either **7** or **P7**) dissociated photochemically, with the rest photodimerizing. Since dimerization is a bimolecular process, its contribution relative to aniline release is suppressed by dilution, but optimizing photorelease conditions was outside the objectives of the current studies.

**Figure 7:**
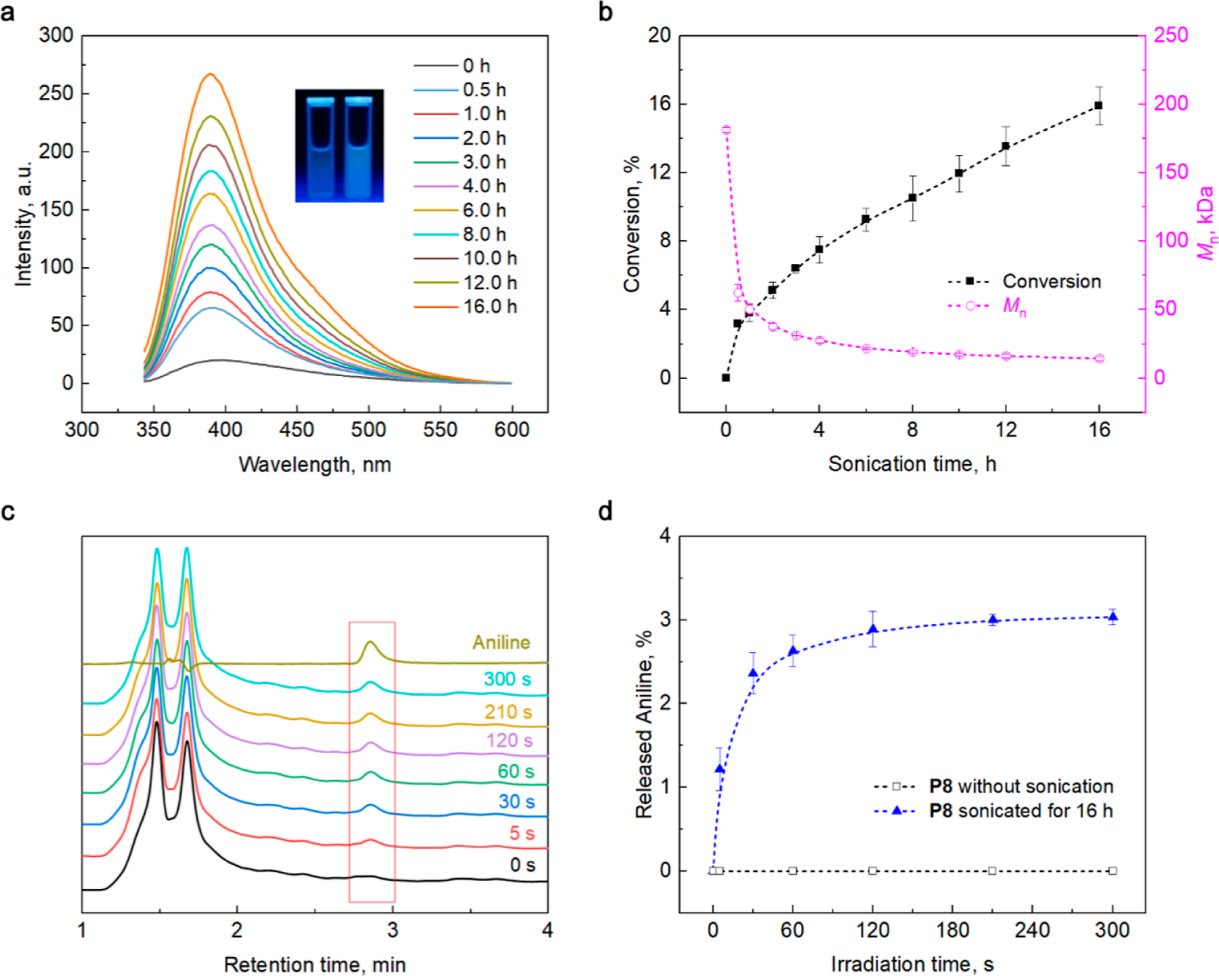
Photomechanochemical properties of **P8**. (a) Fluorescence spectra (λ_exc_ = 322 nm) of a **P8** solution (6.0 mg/mL, 0.033 mM in deionized water) at increasing sonication times. Insets: photograph of the **P8** solutions under UV light (λ_exc_ = 365 nm); before (left) and after (right) sonication. (b) Yield of **P7** and the number-average molar mass, *M*_n_, of a sonicated solution of **P8** as a function of the sonication time. Note that conversion increases more steeply with sonication time at >2 h than *M*_n_ decreases, which probably reflects the much lower force required to dissociate the dimer than to homolyze a C–C or C–O backbone bond. It was previously shown^66^ that shorter polymer chains reach smaller forces during sonication than longer chains, potentially enabling systematic control of mechanochemical fracture selectivity using the chain size. (c) 280 nm output of HPLC of a **P8** solution that was sonicated for 16 h and irradiated at 365 nm for the time shown. (d) Cumulative yield of aniline from **P8** without sonication and after 16 h sonication as a function of the irradiation time.

Under the same conditions, solutions of **P8** or its small-molecule precursor, **8**, generated no detectable amount of aniline (Figures S42–S47). Sonication of a dilute aqueous solution of **P8** under standard conditions^61^ gradually reduced the average size of the dissolved polymer and made the solution increasingly fluorescent (Figure 7). The corresponding emission spectrum (Figures 7a and S48) matched that of **P7** (Figures S49–S52) and allowed the fraction of the coumarin dimer that dissociated mechanochemically to be estimated (Figure 7b). The relationship between this fraction and *M*_n_ of sonicated **P8** (Figure 7b) was similar to that reported previously,^28^ which attributed the relatively low yield of coumarin generation per chain fracture to the majority of initial polymer chains (**P8** here), synthesized by radical polymerization from a bifunctional precursor, having the dimer at a considerable distance away from the chain center.^69^ Such chains are thought to fracture primarily by mechanochemical homolysis of a C–C or C–O backbone bond that is closer to the chain center than the dimer.

Conversely, sonication of a solution of **P7** reduced the average mass of the polymer without changing the fluorescent intensity of the solution (Figures S53 and S54). Sonication of both **P8** and **P7**, yielded a trace amount of aniline (Figures S55 and S56), consistent with previously reported examples of nonmechanochemical hydrolysis of diverse small-molecule organic solutes in sonicated aqueous media,^75,76^ and confirming that the coumarin-aniline construct is mechanochemically inert.

Whereas intact **P8** is photochemically inert under >300 nm light, the sonicated solution of **P8** released a detectable amount of aniline upon irradiation at 365 nm (Figure 7c,d). Just as in intact and sonicated **P7**, photodimerization competes with aniline release in sonicated **P8** (Figure S57). The yield of aniline per **P8** is determined both by the selectivity of mechanochemical fracture of **P8** (i.e., fracture by dissociation of the coumarin dimer vs by homolysis of C–C or C–O backbone bonds) and partitioning of electronically excited coumarin between aniline release and dimerization. Under standard sonication conditions, the mechanochemical selectivity can be increased either by increasing the localization of the dimer at the chain center^66,67^ or by replacing linear chains with brush polymers, with each chain bearing multiple dimer moieties.^61,66^ Additionally, mechanochemical activation in the solid state may be more selective toward dimer fracture compared to sonicated solutions.^30^ Suppressing photodimerization would require reduction of the concentration of irradiated solutions of coumarin-terminated chains. Finally, combining sonication and irradiation would also likely increase the number of aniline moieties released per polymer chain, but these approaches remain to be demonstrated. The seemingly modest yield of aniline per **P8** is sufficient to induce downstream reactions as we illustrate below.

### Demonstration of Photomechanochemically Controlled Gelation

**Figure 8:**
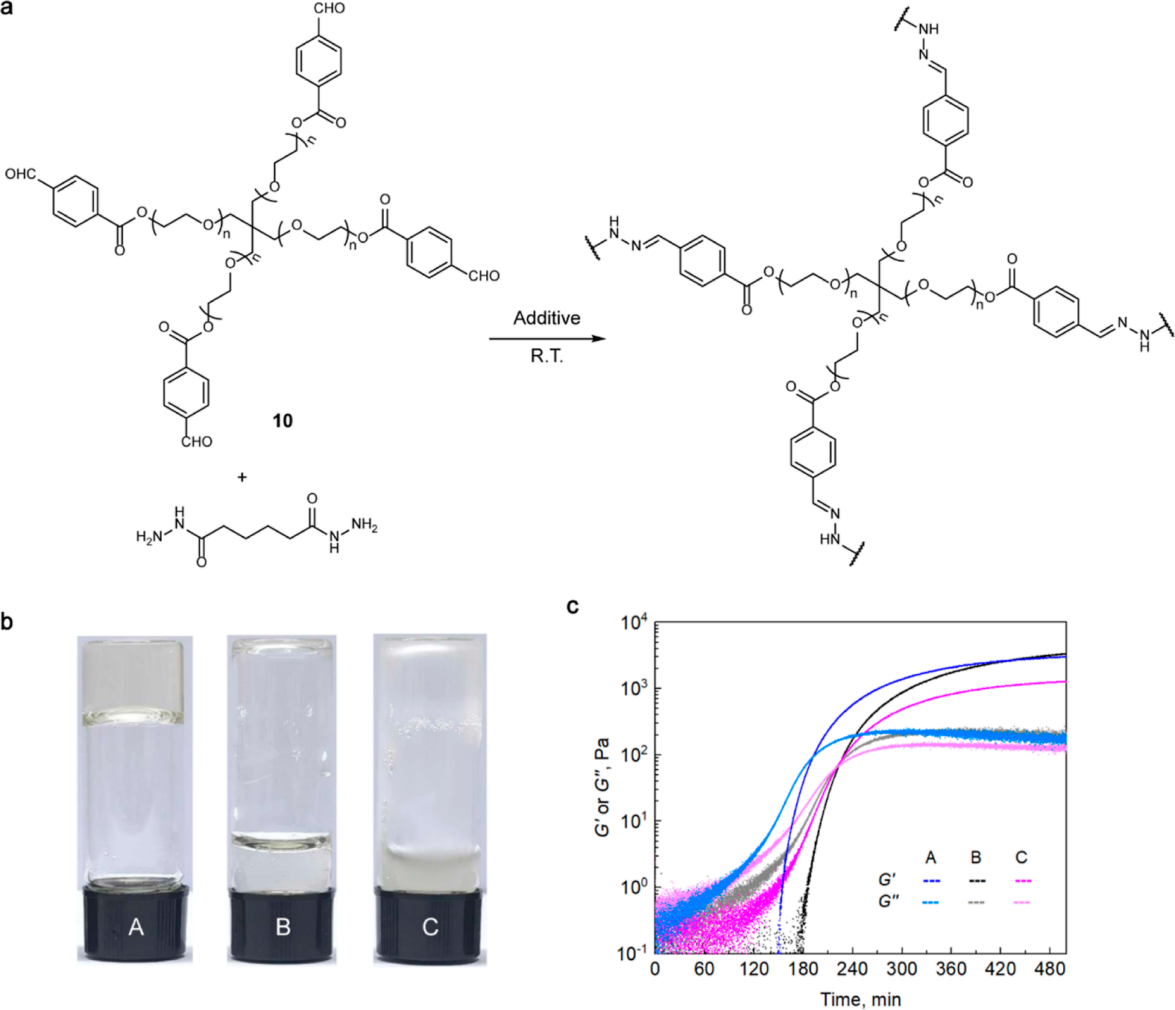
Photomechanically gated gelation by **P8**. (a) Reaction responsible for gel formation. (b) By 210 min after mixing the reagents only the mixture containing sonicated and irradiated **P8** gelled; the remaining samples passed the inverted-vial test by 240 min. (c) Storage and loss moduli, *G*′ and *G*″, respectively, of the mixtures as a function of time since the addition of **P8**; the values are from oscillatory rheology at 0.5% strain and 25 °C.

Whereas the use of photochemically released small molecules to initiate downstream reactions is well established,^1,77^ similar applications of mechanically gated release remain rare.^78^ Here, we demonstrate the capacity of **P8** to catalyze gelation of a mixed aqueous solution of benzaldehyde-terminated 4-arm polyethylene glycol **10** (*M*_n_ = 10 kDa, *D̵*_M_ = 1.02) and adipic dihydrazide in response to sequential mechanical and optical energy input (Figure 8 and Table S3).

We used oscillatory time-sweep rheology to measure time-evolution of the storage and loss moduli, *G*′ and *G*″, respectively, of solutions of **10** and adipic dihydrazide in 0.1 M phosphate buffer containing 100 mg/mL of **P8** that was either sonicated and irradiated (sample A, Table 1), only sonicated but not irradiated (sample B), or neither sonicated nor irradiated (control sample C). All mixtures eventually gel, but sonicated and irradiated **P8** accelerates gelation. Additionally, the moduli of the mixture containing pristine **P8** (sample C) plateau at lower values than those containing treated **P8**, suggesting reduced cross-linking density.^13^ The statistically indistinguishable ultimate *G*′ and *G*″ of samples A and B, combined with the faster gelation of sample A is expected from the detection of trace amounts of aniline in sonicated **P8** prior to irradiation and is consistent with the well-established catalytic effect of aniline on gelation of aldehyde terminated macromers in the presence of a difunctional hydrazide.^79^ The results above demonstrate the potential of photomechanochemical gating to control reactions that are neither mechanochemical nor photochemical. They also indicate that the practical application of this strategy would require optimization of the microstructure of the source of the catalyst (i.e., **P8** here), mechanical loading scenarios (e.g., sonicated solution or sheared melts), and potentially the catalyst. Conversely, our calculations suggest that other isomers of **6** are unlikely to improve the yield under sonication because they have less favorable mechanochemical kinetics than **8**.

**Table 1:** Summary of the Rheological Studies of Gelation of Samples Resulting from Different Pretreatments of **P8**^a^

sample	A	B	C
sonication time, h	16	16	0
irradiation time, min	5	0	5
gelation onset, min	190	220	222
ultimate *G*′, kPa	3.0	3.1	1.2
ultimate *G*″, kPa	0.18	0.18	0.12

a Time after mixing all reagents when *G*′ first exceeded *G*″.

## Conclusions

We introduced the concept of a photomechanochemical AND gate and demonstrated it experimentally with a coumarin dimer that releases aniline only in response to sequential application of a mechanical load and irradiation at 365 nm. The dimer is transparent to light of >300 nm, where it is photochemically inert, but dissociates rapidly under extrinsic tensile force. The resulting coumarin releases any moiety bound to its cyclobutane hydroxymethyl groups under 365 nm irradiation. Our choice of aniline as the released payload enabled us to demonstrate photomechanochemically controlled gelation as an illustration of potential applications of this idea. Among many plausible implementations of a photomechanochemical AND gate, our choice of the coumarin dimer was motivated by the results of our detailed DFT calculations of structure–mechanochemical reactivities. These calculations revealed a remarkable diversity of the kinetics and mechanisms of dissociation of simple, synthetically readily accessible, derivatives of coumarin dimers. Our results suggest that, in addition to photomechanochemical gating explored in this paper, such dimers may be better suited for practical exploitation of mechanochemistry than other mechanophores reported to date for, mechanochromism, mechanofluoresence,^11^ optical healing of mechanical degradation,^80^ and balancing the stability of polymers during their working lifetime and the ease of recycling them, two desirable properties that are often in competition.^2,4^ For example, the high thermal stability of the studied coumarin dimers in the absence of a load makes them compatible with varied polymer processing methods,^81^ while the simplicity of tuning the force sensitivity of the dissociation kinetics by substitution facilitates the molecular design of a coumarin dimer for each of the very diverse loading conditions that have been identified in practical manifestations of mechanochemistry.^10,38,67,82^ The reported force-dependent kinetics and mechanisms enable such a perspective exploration of coumarin dimers for fundamental and applied polymer mechanochemistry.

## Supporting Information Available

DFT calculations, general experimental information, synthetic procedures, characterization data of the products, and HPLC, absorbance, and other physical measurements demonstrating the photomechanochemical gating concept (PDF)
Cartesian coordinates of the minimum-energy conformers of all kinetically significant stationary states, and the free energies of these states as a function of the applied force (ZIP)
