# Supplementary material for: Coumarin Dimer Is an Effective Photomechanochemical AND Gate for Small-Molecule Release
Source: J Am Chem Soc. 2023 Oct 11;145(42):23214–26. doi: 10.1021/jacs.3c07883 (PMC10603814; doi:10.1021/jacs.3c07883)
Supplement: Supplementary file 1 — ja3c07883_si_001.pdf [file ja3c07883_si_001.pdf]

Supporting Information

A coumarin dimer is an effective photomechanochemical AND gate for small-molecule release

Xiaojun He[1ǂ], Yancong Tian[2ǂ], Robert T. O'Neill[2], Yuanze Xu[1], Yangju Lin*[1], Wengui Weng*[1], Roman Boulatov*[2]

[1] Department of Chemistry, College of Chemistry and Engineering, Xiamen University, Xiamen, Fujian 361005, China

[2] Department of Chemistry, University of Liverpool, Crown St., Liverpool, L69 7ZD UK.

ǂ equal contribution

Contents

I. DFT calculations S2

II. General experimental Information S4

III. Method S4

1. Ultrasound sonication experiments S4
2. UV Irradiation experiments S5

IV. Small molecule synthesis S5

7-(2-hydroxyethoxy)-4-methyl-2*H*-chromen-2-one (**S1**) S6

2-((4-methyl-2-oxo-2*H*-chromen-7-yl)oxy)ethyl 2-bromo-2-methylpropanoate (**S2**) S7

2-((4-formyl-2-oxo-2*H*-chromen-7-yl)oxy)ethyl 2-bromo-2-methylpropanoate (**S3**) S9

2-((4-(hydroxymethyl)-2-oxo-2*H*-chromen-7-yl)oxy)ethyl 2-bromo-2-methylpropanoate (**9**) S11

Phenyl isocyanate (**S4**) S14

2-((2-oxo-4-(((phenylcarbamoyl)oxy)methyl)-2H-chromen-7-yl)oxy)ethyl 2-bromo-2-methylpropanoate (**7**) S14

(((6a*S*,6b*S*,12b*R*,12c*R*)-6,7-dioxo-12b,12c-bis(((phenylcarbamoyl)oxy)methyl)-6,6a,6b,7,12b,12c-hexahydrocyclobuta[1,2-c:4,3-c']dichromene-3,10-diyl)bis(oxy))bis(ethane-2,1-diyl) bis(2-bromo-2-methylpropanoate) (**8**) S16

V. Polymer Synthesis S19

VI. Physical measurements, material characterization and data processing S22

1. Estimation of Mechanochemical Activation Yield S22
2. Photochemical release of aniline. S23
3. Photomechanically controlled gelation. S37

VII. References S39

I. DFT calculations

All calculations were performed with the Gaussian 16 suite of software in vacuum. The UHF formalism was applied to all open-shell singlets (TS_1, Int and TS_2) and the wavefunction stability checks were performed on all converged geometries of TS_1, Int and TS_2; on all initial guess structures of the transition states and on all converged geometries of the dimer coupled to force >4 nN. The Berny algorithm was applied to locate stationary points. The nature of each converged strain-free geometry and select force-coupled geometries was confirmed by the presence of 0 (minima) or 1 (saddle points) imaginary frequencies obtained by analytical frequency calculations. Tight convergence criteria and ultrafine integration grids were used in optimisations and frequency calculations. All force-coupled stationary geometries were optimized as previously described.[1] Force dependent electronic energies, constrained distances and thermodynamic corrections (see below) were obtained by interpolation of the results of relaxed potential energy scans of either the terminal ${}_{MeO}C\cdots C_{OMe}$ (series 2, 4-6) or terminal ${}_{H}C\ldots C_{H}$ (series 3) distance of the dimer.

The initial (guess) geometry of the 1st dissociation transition state of each unsubstituted coumarin dimer was generated by scanning, at uMPW1K/6-31+G(d), the proximate scissile bond (hh isomers) or any scissile bond (ht isomers) of the cyclobutene core, identifying the converged geometry corresponding to the highest energy, performing wavefunction stability test, frequency calculation, unconstrained optimization to a saddle point, another wavefunction stability test and final frequency. The initial guess geometry of TS_2 was derived for each conformer of Int by scanning the remaining scissile bond. Conformers of Int were generated systematically as previously described.[2] Guess geometries of transition states of the alternative dissociation path (distal bond scission followed by proximate bond scission, Fig. 2b main text) were generated by manually elongating the scissile bond of the dimer (TS_{1a}) or of each conformer of Int (TS_{2a}) to the value of the scissile bond in the corresponding converged conformer of TS_1 or TS_2. The initial guess geometries of the concerted TS_s (ht isomers) were obtained by manually elongating both scissile bonds of the cyclobutane core to 1.85 Å.

Thermodynamic corrections (*TC*s) to electronic energies of individual converged geometries were calculated statistical-mechanically in the pseudo-harmonic oscillator/rigid rotor/ideal gas approximations, as $3RT + ZPE + U_{vib} - TS$, where ZPE is the zero-point energy, U_{vib} is the vibrational component of the internal energy and S is the total entropy. Vibrational frequencies below 500 cm^{-1} were replaced with 500 cm^{-1} as previously recommended,[3] to avoid the artifactually high contribution of such low-frequency modes to the vibrational entropy. The use of analytical frequencies calculated on converged force-coupled geometries in this study is theoretically sound because the calculation is performed on the molecule plus its infinitely-compliant constraint (rather than just the molecule), which is a stationary point with all internal forces at 0.[4,5]

Table S1. The standard free energies (kcal/mol) of the kinetically significant stationary states of dissociation of the 4 isomeric unsubstituted coumarin dimers with 4 functionals and 6-31+G(d) basis set in vacuum. The rate-limiting TS is in bold. The UHF designation is omitted for brevity.

**isomer**	**Stationary state**	**MPW1K**	**CAM-B3LYP**	**MPWKCIS1K**	**BMK**
hh-anti	TS_1	35.1	**35.2**	**36.5**	41.2
	Int	32.3	32.3	33.9	39.7
	TS_2	**35.4**	34.0	36.1	**45.3**
hh-syn	TS_1	**38.0**	**37.8**	**39.2**	**44.8**
	Int	27.5	27.0	28.6	35.3
	TS_2	33.0	31.1	33.1	36.8
ht-anti	TS_1	**40.5**	**40.5**	**42.1**	47.3
	Int	39.4	39.0	40.5	46.3
	TS_2	40.5	39.2	41.2	**49.5**
ht-syn	TS_1	**41.3**	**42.8**	**42.8**	**48.6**
	Int	37.5	36.5	36.4	42.8
	TS_2	39.6	39.3	39.9	44.3

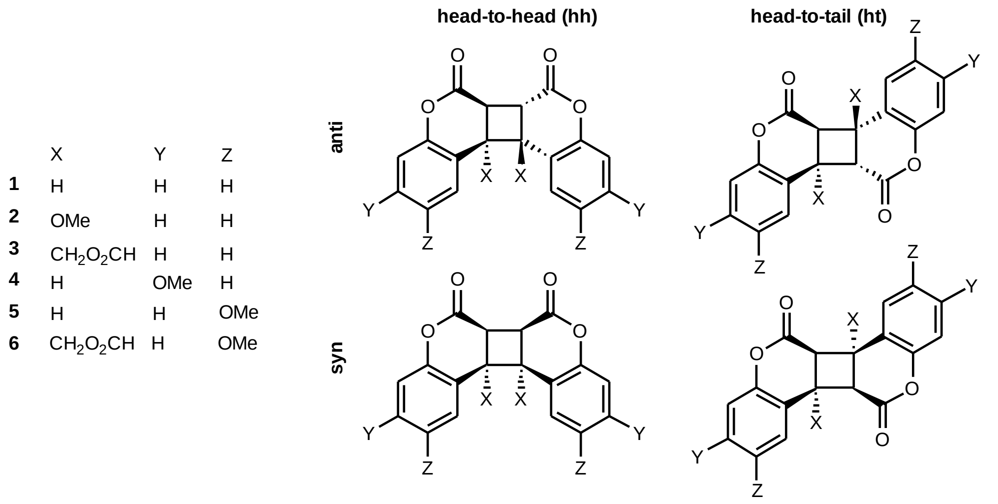

Table S2. Maximum force in nN at which the R/TS_1 and Int/TS_2 pairs exist.

Series	hh-anti		hh-syn		ht-anti		ht-syn	
**2**	>5.5	2.4	4.0	2.8	>5.5	2.3	>5.5	2.3
**3**	>5.5	1.3	4.0	2.9	>5.5	2.4	>5.5	2.3
**4**	4.6	>5.5	3.1	5.2	>5.5	4.4	>5.5	4.9
**5**	3.2	>5.5	3.0	>5.5	>5.5	4.5	>5.5	4.7

$$\Delta G_d^{\ddagger} = \Delta G_1^{\ddagger} + RTln\left(1 + e^{\frac{\Delta G_2^{\ddagger} - \Delta G_1^{\ddagger}}{RT}}\right) \qquad eq.S1$$

Cartesian coordinates of conformational minima of the strain-free kinetically significant states of coumarin dimers **1-6**, including with multiple functionals, and their force-dependent free energies are

tabulated in suppdata.mat.

II. General experimental Information

All reagents and solvents from commercial suppliers were used without further purification unless otherwise stated. 7-hydroxy-4-methyl-2*H*-chromen-2-one, selenium dioxide and copper wire (diameter = 0.64 mm) were purchased from Alfa Aesar. 2-iodoethanol, α-bromoisobutyryl bromide, triethylamine, triphosgene, dibutyltin dilaurate, Me_6TREN, *N,N*-dimethylformamide, NH_4BF_4, dry acetonitrile, dry dichloromethane and dry DMSO were obtained from Energy Chemical. Oligo(ethylene glycol) methyl ether methacrylate (OEGMA, M_n = 300) was supplied from Sigma-Aldrich and 4-arm-PEG-OH was provided by Xiamen SINOPEG. Other reagents and solvents were purchased from Sinopharm.

^{1}H NMR and ^{13}C NMR spectra were recorded in $CDCl_3$ or DMSO-d_6 or D_2O or DMF-d_7 at 25 ℃ on a Bruker Avance III spectrometer (500 MHz 1H) or Bruker Avance II spectrometer (400 MHz 1H). The chemical shifts were given in ppm (δ) based on internal TMS or residual protonated solvent. The peak patterns are indicated as follows: s, singlet; d, doublet; t, triplet; q, quartet; m, multiplet; qui, quintet; sxt, sextet. The coupling constants, J are reported in Hertz (Hz). Mass spectroscopy data were collected on an autoflex maX MALDI-TOF MS instrument. The UV-Vis spectroscopy was performed on Thermo Scientific Multiskan SkyHigh and fluorescence spectra on F7000. Ultrasound experiments were carried out on a Sonics Vibra-Cell 505 liquid processor with a 12.8 mm (diameter) titanium solid probe. Gel permeation chromatography (GPC) was executed using Waters GPC system equipped with 2414 differential refractometer and four columns (7.8 × 300 mm). The eluent was *N,N*-dimethylformamide (DMF) (HPLC grade, ⩾ 99.9%) containing 5.0 mM NH_4BF_4. HPLC was recorded by Aglilent LC-1200 equipped with an Eclipse XDB-C18 column and a UV-vis detector monitored at 280 nm (gradient :0 min, 50% CH_3OH - 50% H_2O; 4 min, 60% CH_3OH - 40% H_2O; 10 min, 100% CH_3OH; flow 1.0 mL/min). Rheology test was performed on a TA discovery hybrid rheometer equipped with a 25 mm parallel plate and a ETC environmental control chamber at room temperature.

III. Method

1. Ultrasound sonication experiments

The sonication experiments were carried out on a Sonics Materials Vibra Cell 505 liquid processor equipped with a 12.8 mm (diameter) titanium solid probe using aqueous solutions of polymers at 6.0 mg/mL (0.033 mM) in a 3-neck glass cell immersed in an ice-water bath. Before sonication, the solution was purged with Ar for 20 min. Total experiments were carried out under Argon with a pulse sequence of 1.0 s on followed by 1.0 s off at a nominal power of 1.98 W/cm^2 (f = 20 kHz, 30% amplitude, E = 147361 J, t = 57600 s, A = 1.29 cm^2). The temperature of the system was kept at 0-5 ^{o}C. 0.6 mL or 1.0 mL solution were withdrawn periodically from the cell for UV-vis, fluorescence and GPC tests.

2. UV Irradiation experiments

The irradiation experiments were performed on a COUSZ LED UV light equipped with a standard focusing mirror UPUL008 (λ = 365 nm, 6500 mW/cm^2) at room temperature. 300 μL solution containing **P7** (6.0 mg/mL, 0.044 mM), **P8** (6.0 mg/mL, 0.033 mM), sonicated **P8** in H_2O (6.0 mg/mL, 0.033 mM), **7 (**0.05 mM), or **8** in DMF/H_2O (2:1 by volume, 0.10 mM) was filled into a quartz cuvette (2 mm × 10 mm), which was then irradiated with UV light. The UV light was placed vertically at a distance of 1 cm from the top of container, and irradiation time range from 5 to 300 s with a step time in between 5 - 30 s. After each irradiation step, the UV-vis absorption spectra were recorded.

For NMR tests, the concentration of small molecule was 0.4 mM in DMF-d_7/D_2O (volume ratio of 2/1, 0.5 mL) and it was 200 mg/mL in D_2O (0.5 mL) for polymer. The irradiation process was performed in an NMR tube covered by alumina foil. Due to the considerable overlap of NMR signals between reactant and product, the yield of photochemically released aniline was measured by HPLC. Generally, 1.8 mL solution containing **P7** (6.0 mg/mL, 0.044 mM), **P8** (6.0 mg/mL, 0.033 mM), sonicated **P8** in H_2O (6.0 mg/mL, 0.033 mM), **7** (10 μM) or **8** in 50% aqueous CH_3OH (15 μM) was filled into a quartz cuvette (10 mm × 10 mm) and irradiated with UV light. After each irradiation step, the sample was analysed by HPLC equipped with a UV-vis detector. The signal at 280 nm was monitored, and the generated aniline was quantified using a calibration curve.

IV. Small molecule synthesis

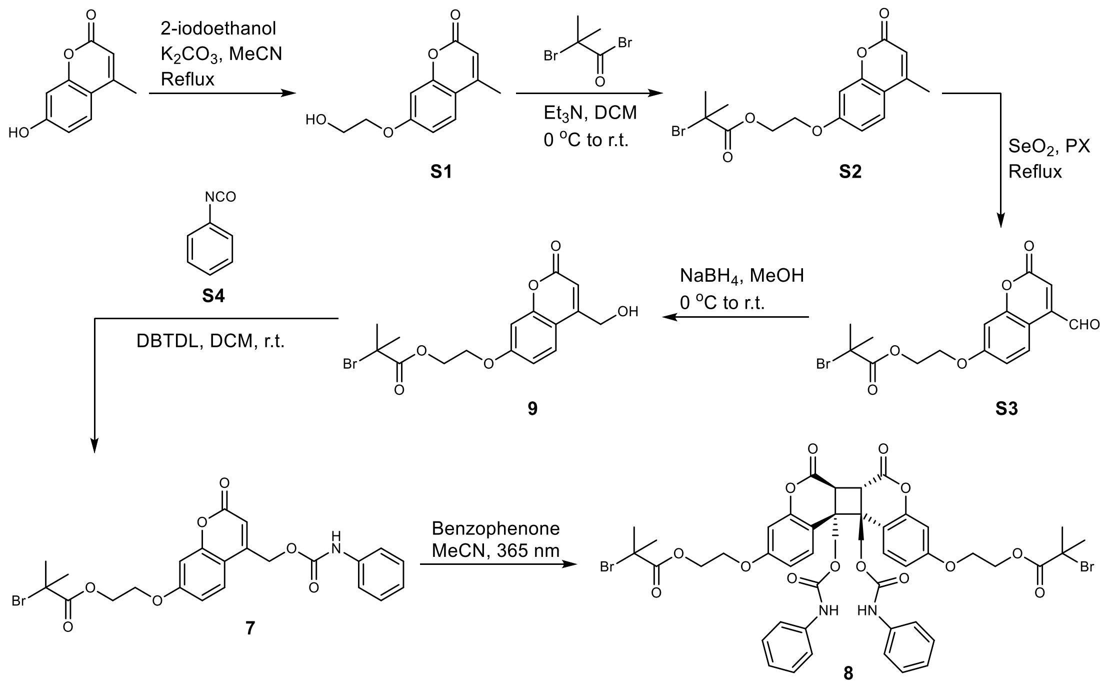

Figure S1. Syntheses of coumarin **7** and its dimer, **8**.

7-(2-hydroxyethoxy)-4-methyl-2*H*-chromen-2-one (**S1**)

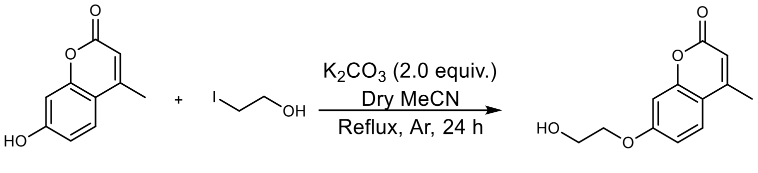

S1

Figure S2. Synthetic route of 7-(2-hydroxyethoxy)-4-methyl-2*H*-chromen-2-one (**S1**).

The synthesis was adapted from the literature[6]. 7-hydroxy-4-methyl-2*H*-chromen-2-one (4.4043 g, 25.0 mmol) and anhydrous potassium carbonate (6.9105 g, 50.0 mmol) were suspended in dry acetonitrile (50 mL) in a 100 mL round-bottom flask followed by dropwise addition of 2-iodoethanol through syringe. The reaction was refluxed and stirred under argon for 24 h. After completion, the mixture was filtered in hot and washed with dichloromethane to remove the starting material. The filtrate was evaporated under reduced pressure to give a white crude product **S1** (4.7080 g, 85.5%). ^{1}H NMR (500 MHz, $CDCl_3$) δ 7.49 (d, J = 9.0 Hz, 1H), 6.88 (dd, J = 8.5 2.5 Hz, 1H), 6.82 (d, J = 5.0 Hz, 1H), 6.13 (s, 1H), 4.14 (t, J = 4.5 Hz, 2H), 4.00 (t, J = 4.5 Hz, 2H), 3.73 (s, 1H), 2.39 (s, 1H); ^{13}C NMR (125 MHz, $CDCl_3$) δ 161.7, 155.1, 125.7, 115.0, 113.9, 112.6, 112.1, 112.0, 101.6, 69.8, 61.1, 18.7.

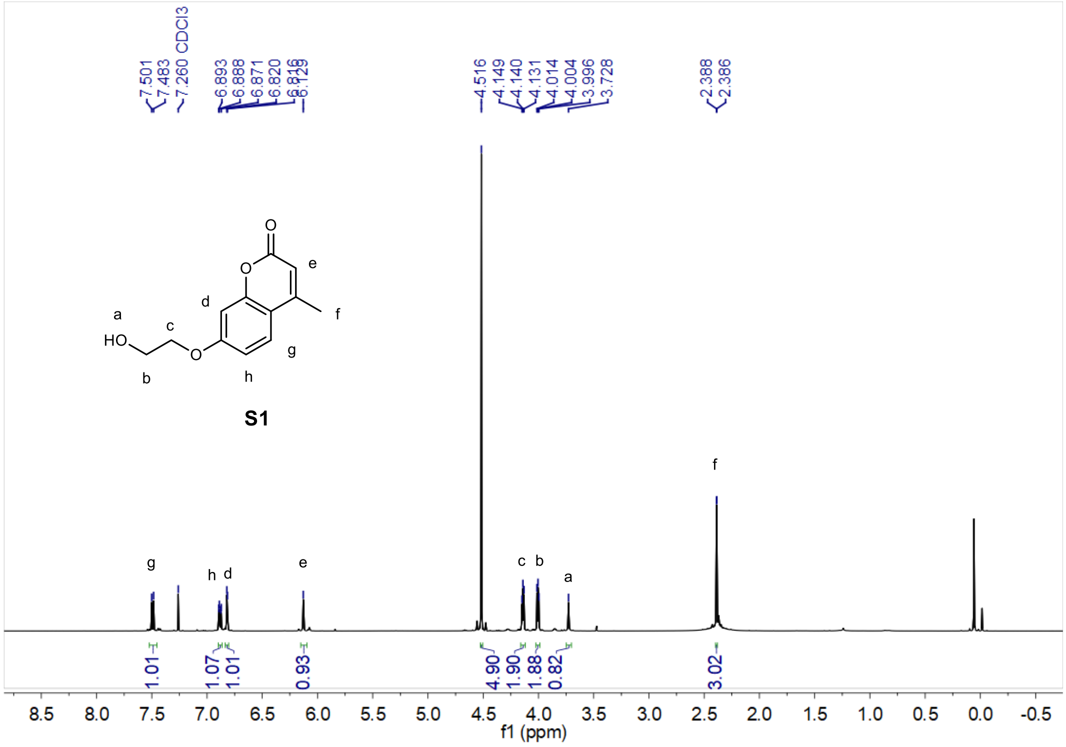

Figure S3. ^{1}H NMR spectrum of compound **S1**.

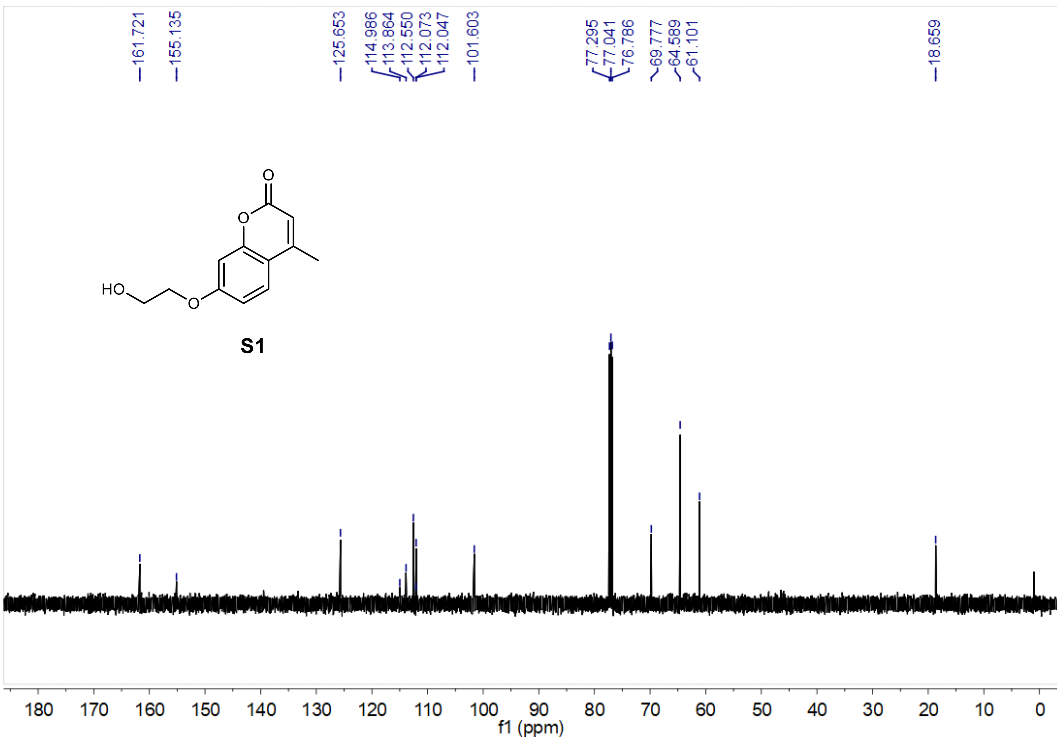

Figure S4. ^{13}C NMR spectrum of compound **S1**.

2-((4-methyl-2-oxo-2*H*-chromen-7-yl)oxy)ethyl 2-bromo-2-methylpropanoate (**S2**)

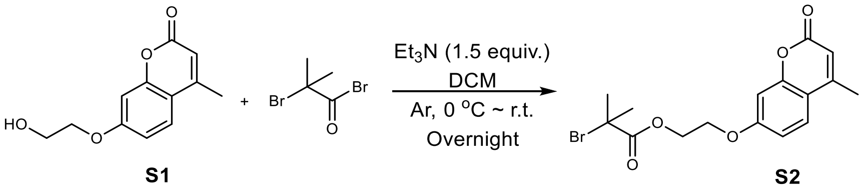

Figure S5. Synthetic route of 2-((4-methyl-2-oxo-2*H*-chromen-7-yl)oxy)ethyl 2-bromo-2-methylpropanoate (**S2**).

Compound **S1** (4.7080 g, 21.4 mmol) and triethylamine (3.2482 g, 32.1 mmol) were dissolved in dichloromethane (60 mL) in a 150 ml round-bottom flask charged with argon, followed by dropwise addition of α-bromoisobutyryl bromide (7.3724 g, 32.1 mmol) through syringe at 0 ℃. The resulting mixture was moved to room temperature and stirred overnight. After starting material **S1** was consumed (monitored by TLC), the reaction was quenched by saturated sodium bicarbonate (50 mL) and extracted with dichloromethane three times (50 mL × 3). The organic solution was combined and washed with saturated brine, dried over anhydrous sodium sulfate, filtered, and evaporated in vacuo. The residue was purified by column chromatography using dichloromethane as eluent to afford a white solid **S2** (3.6069 g, 45.7%). ^{1}H NMR (500 MHz, $CDCl_3$) δ 7.49 (d, J = 9.0 Hz, 1H), 6.87 (dd, J = 8.5 2.5 Hz, 1H), 6.80 (d, J = 2.5 Hz, 1H), 6.11 (s, 1H), 4.54 (t, J = 4.8 Hz, 2H), 4.27 (t, J = 4.8 Hz, 2H), 2.38 (s, 3H), 1.92 (s, 6H); ^{13}C NMR (125 MHz, $CDCl_3$) δ 171.5, 161.3, 161.0, 155.1, 152.4, 125.6,

113.9, 112.4, 112.1, 101.6, 66.0, 63.6, 55.3, 30.6, 18.6. MALDI-TOF-MS *m/z* calculated for $C_{16}H_{17}BrO_5$ $[M+H]^+$: 369.033; Found: 369.032.

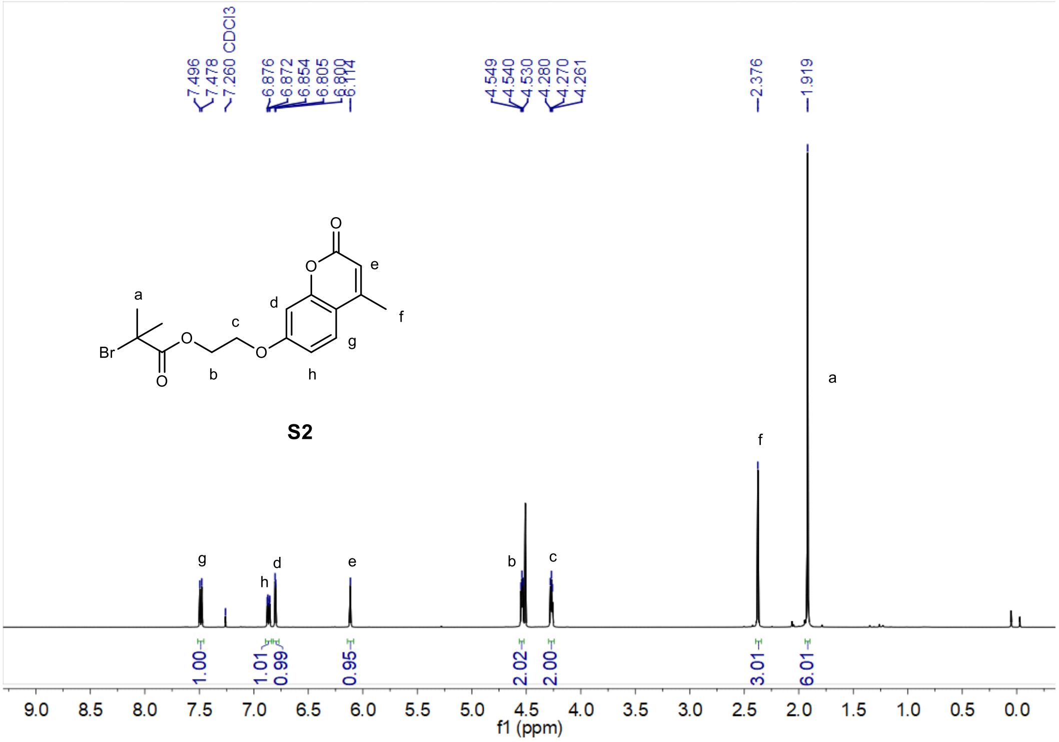

Figure S6. ^{1}H NMR spectrum of compound **S2**.

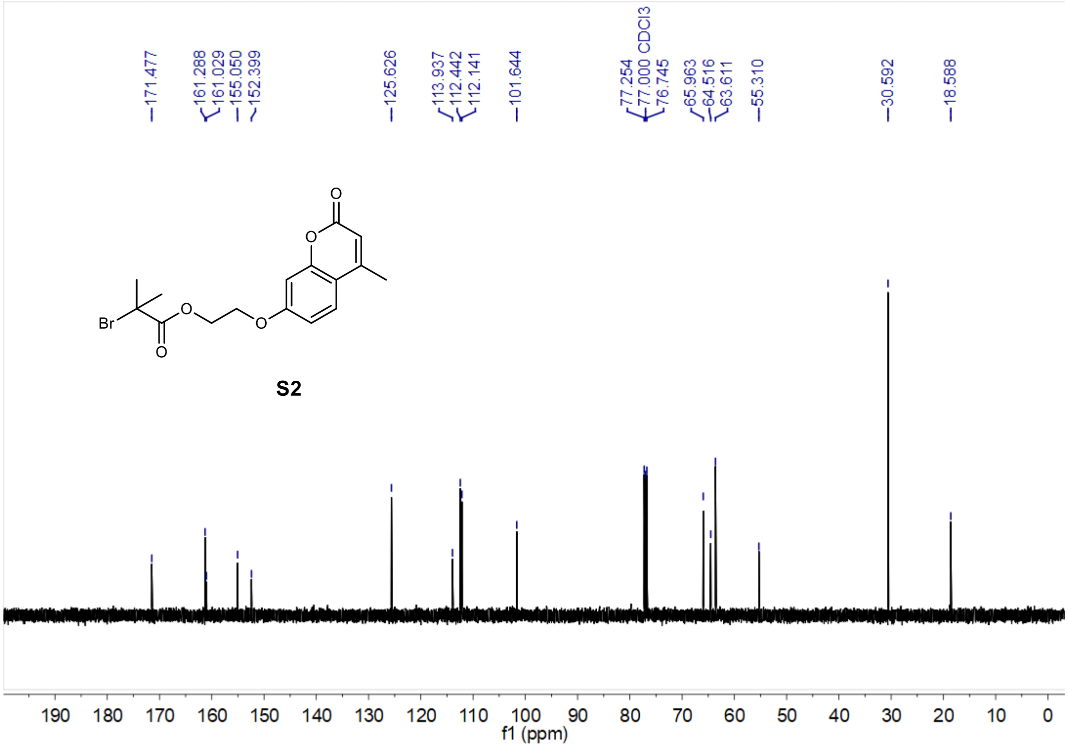

Figure S7. ^{13}C NMR spectrum of compound **S2**.

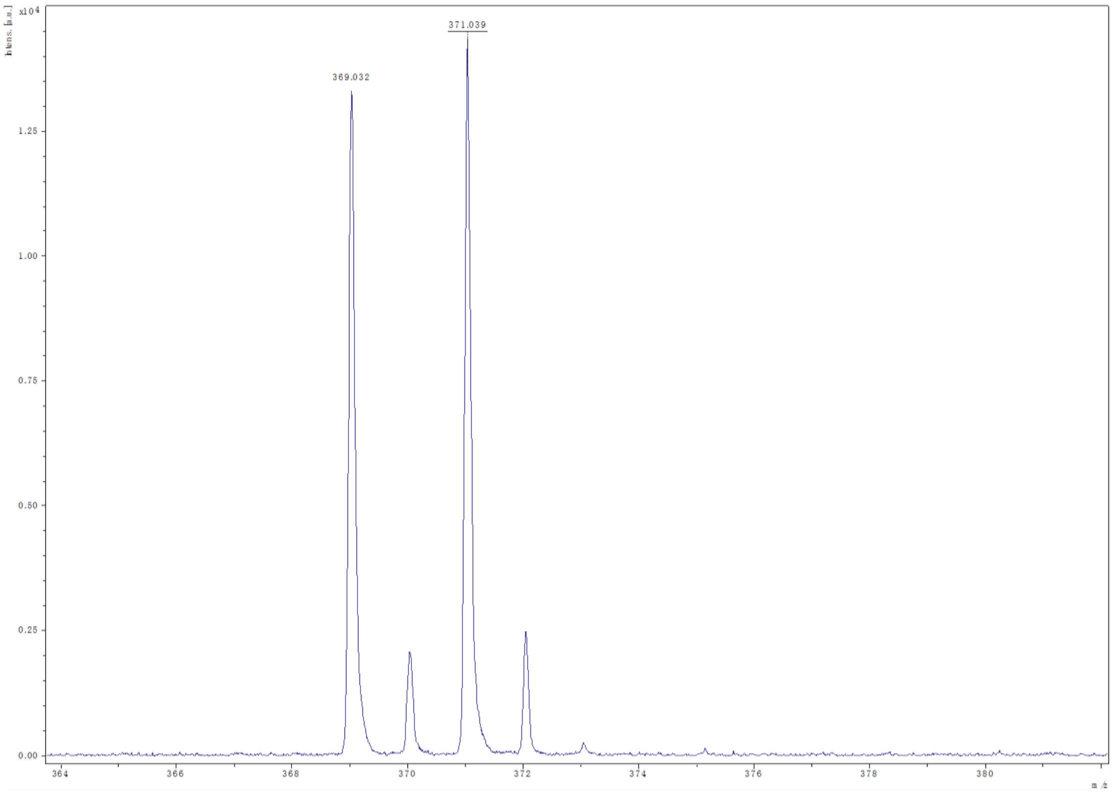

Figure S8. MALDI-TOF-MS spectrum of **S2** (*m/z* calculated for $C_{16}H_{17}BrO_5$ $[M+H]^+$: 369.033; Found: 369.032).

2-((4-formyl-2-oxo-2*H*-chromen-7-yl)oxy)ethyl 2-bromo-2-methylpropanoate (**S3**).

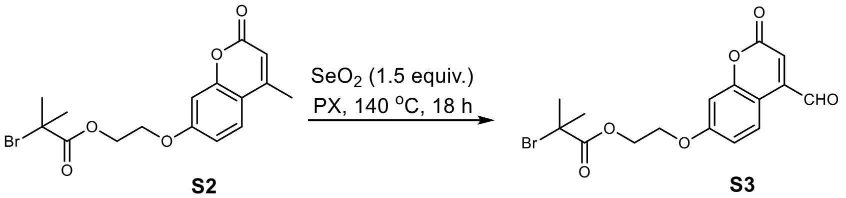

Figure S9**.** Synthetic route of 2-((4-formyl-2-oxo-2*H*-chromen-7-yl)oxy)ethyl 2-bromo-2-methylpropanoate (**S3**).

The synthesis was adapted from the literature[7]. 2-((4-methyl-2-oxo-2*H*-chromen-7-yl)oxy)ethyl 2-bromo-2-methylpropanoate **S2** (3.6069 g, 9.77 mmol) and selenium dioxide (1.6260 g, 14.7 mmol) were suspended in *p*-xylene (30 mL) in a 100 mL round-bottom flask. The reaction was refluxed and stirred for 18 h. After completion, the mixture was filtrated without cooling to room temperature and the residue was washed with dichloromethane. The filtrate was evaporated in vacuo to give a crude product. Purification by column chromatography in dichloromethane afforded the desired product **S3** as a yellow solid (3.1230 g, 83.4%). ^{1}H NMR (500 MHz, $CDCl_3$) δ 10.1 (s, 1H), 8.49 (d, J = 9.0 Hz, 1H), 6.93 (dd, J = 6.5 2.5 Hz, 1H), 6.87 (d, J = 2.5 Hz, 1H), 6.72 (s, 1H), 4.56 (t, J = 4.8 Hz, 2H), 4.30 (t, J = 4.8 Hz, 2H), 1.93 (s, 6H); ^{13}C NMR (125 MHz, $CDCl_3$) δ 191.6, 171.5, 162.0, 160.5, 156.3, 143.6,

127.5, 122.5, 113.4, 108.6, 101.9, 66.1, 63.5, 55.2, 30.6. MALDI-TOF-MS *m/z* calculated for $C_{16}H_{15}BrO_6$ $[M+H]^+$: 383.012; Found :383.011.

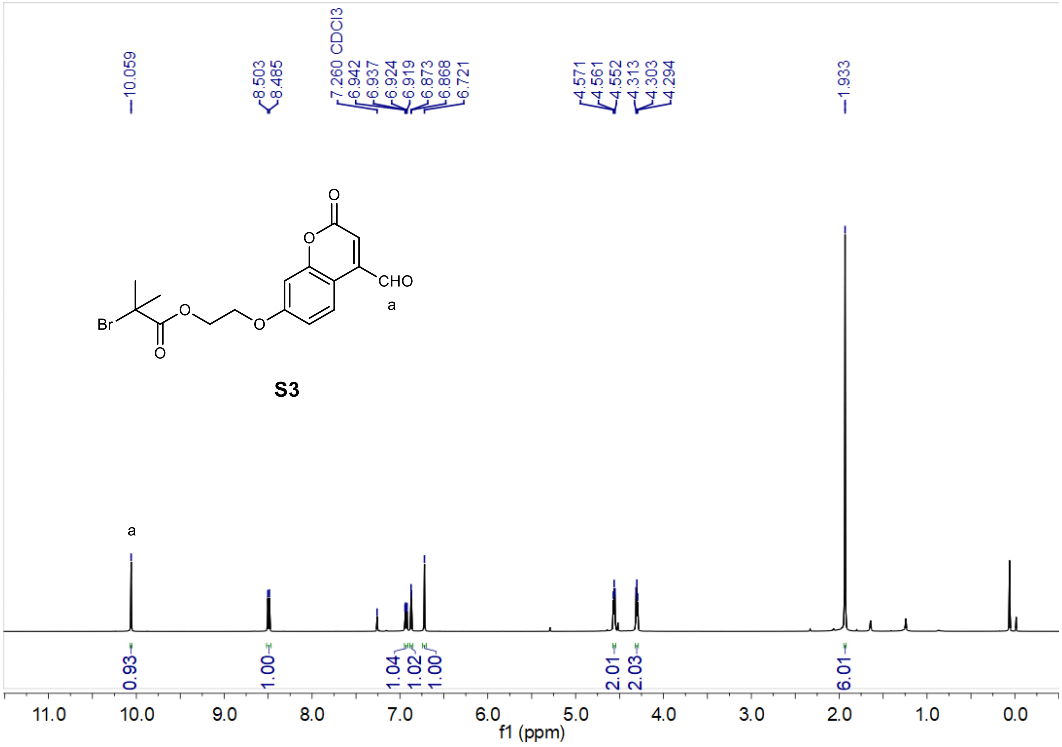

Figure S10. ¹H NMR spectrum of compound **S3**.

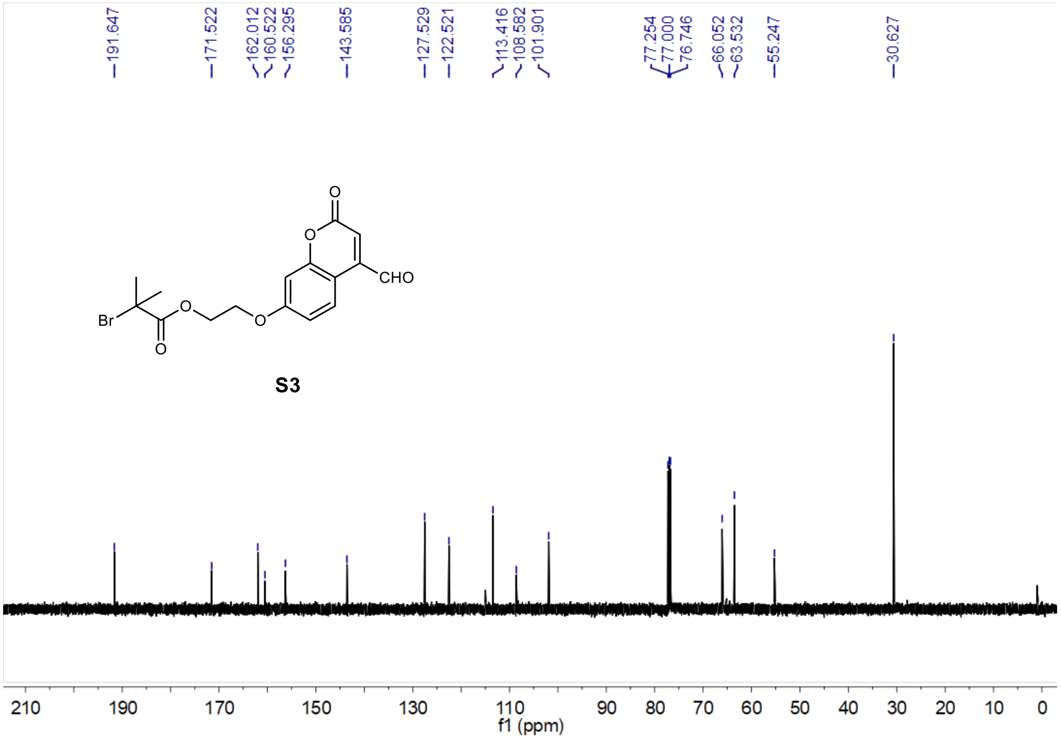

Figure S11. ¹³C NMR spectrum of compound **S3**.

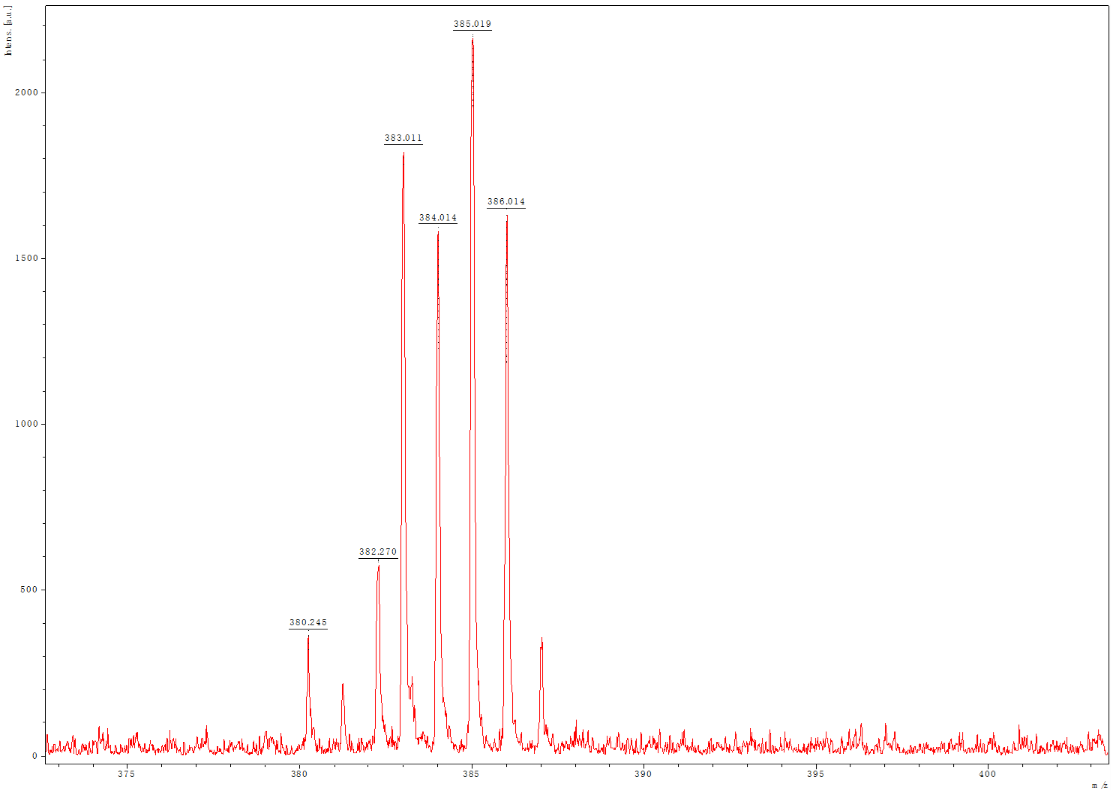

Figure S12. MALDI-TOF-MS spectrum of **S3** (*m/z* calculated for $C_{16}H_{15}BrO_6$ $[M+H]^+$: 383.012; Found :383.011).

2-((4-(hydroxymethyl)-2-oxo-2*H*-chromen-7-yl)oxy)ethyl 2-bromo-2-methylpropanoate (**9**)

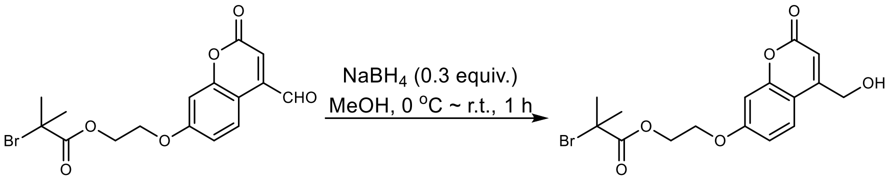

Figure S13. Synthetic route of 2-((4-(hydroxymethyl)-2-oxo-2*H*-chromen-7-yl)oxy)ethyl 2-bromo-2-methylpropanoate (**9**).

(2-((4-formyl-2-oxo-2*H*-chromen-7-yl)oxy)ethyl 2-bromo-2-methylpropanoate **S3** (1.8512 g, 4.83mmol) was dissolved in methanol (40 mL) in a 100 mL round-bottom flask. The solution was cooled to 0 ~ 5 °C under ice bath, and sodium borohydride (54.8 mg, 1.45mmol) was added. After stirring at room temperature for 1 h, the reaction was quenched by deionized water (40 mL) and neutralized with 1.0 M HCl. The obtained mixture was extracted with ethyl acetate three times (50 mL × 3), and the combined organic layer was dried over anhydrous sodium sulfate, filtrated, evaporated under reduced pressure. The residue was purified by column chromatography (dichloromethane : methanol = 100 :4) to afford a white solid **9** (1.0677 g, 57.4%). ^{1}H NMR (500 MHz, DMSO-d_6) δ 7.61 (d, J = 9.0 Hz, 1H), 7.04 (d, J = 2.0

Hz, 1H), 6.95 (dd, J = 9.0 2.5 Hz, 1H), 6.31 (s, 1H), 5.61 (t, J = 5.5 Hz, 1H), 4.73 (d, J = 4.5 Hz, 2H), 4.50 (t, J = 4.2 Hz, 2H), 4.36 (t, J = 4.5 Hz, 2H), 1.88 (s, 6H); ^{13}C NMR (125 MHz, DMSO-d_6) δ 171.2, 161.5, 160.9, 157.0, 155.2, 125.9, 113.0, 111.4, 108.1, 102.0, 66.7, 64.4, 59.6, 57.4, 30.7. MALDI-TOF-MS m/z calculated for $C_{16}H_{17}BrO_6$ $[M+H]^+$: 385.028; Found: 385.027.

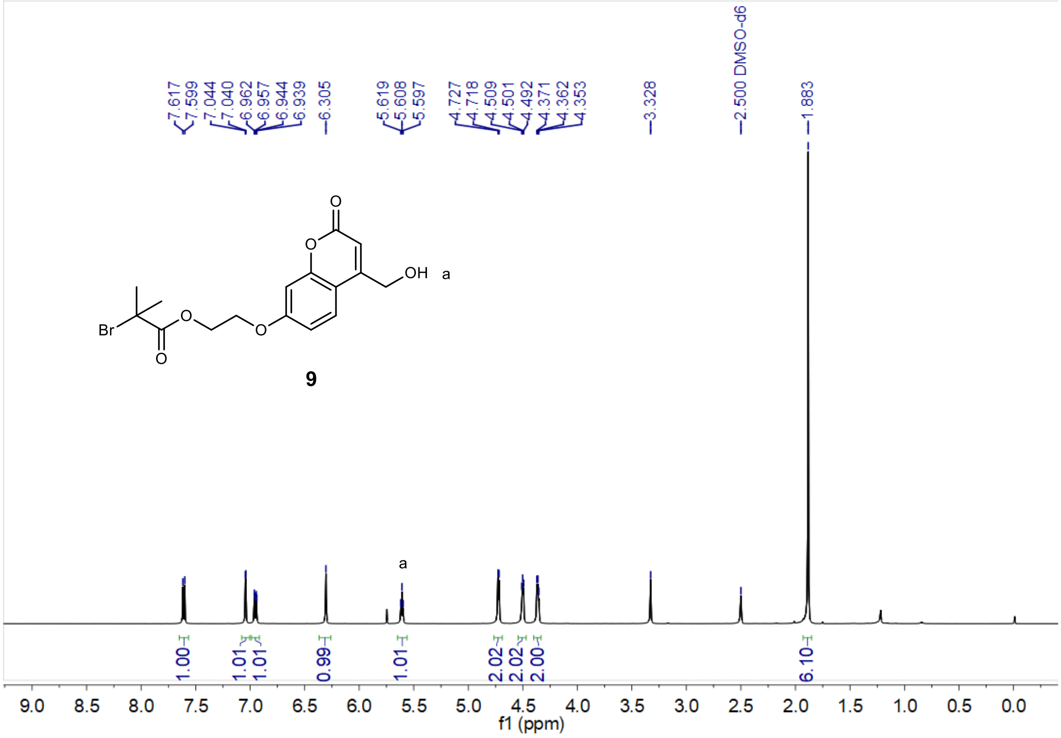

Figure S14. ^{1}H NMR spectrum of compound **9**.

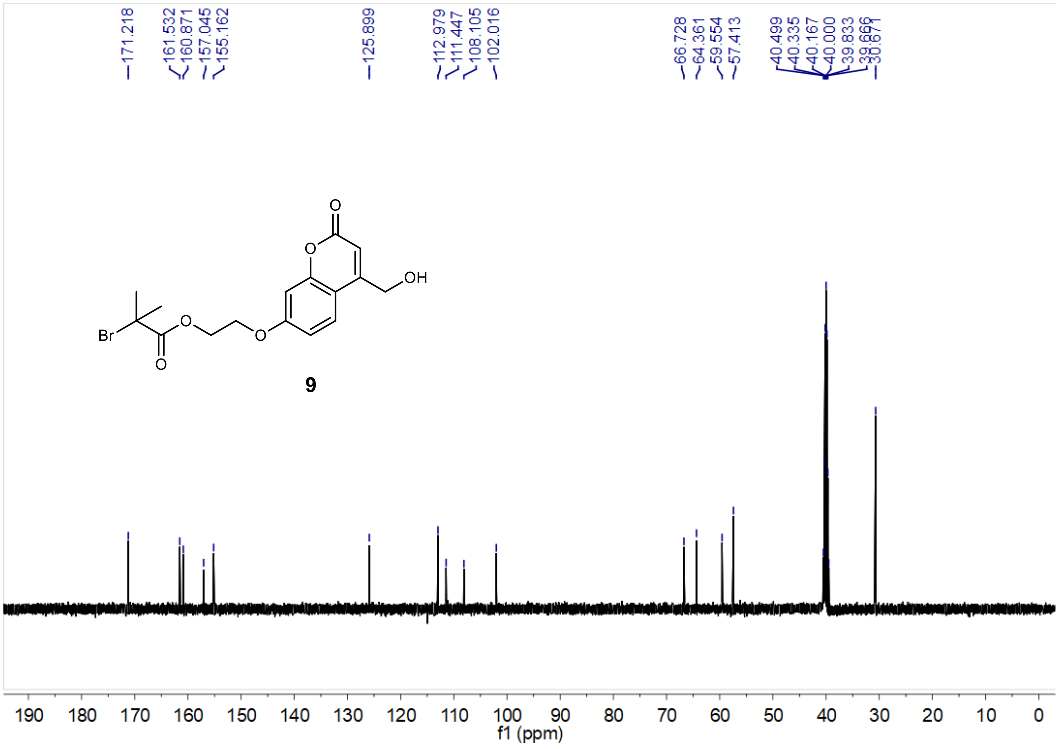

Figure S15. ^{13}C NMR spectrum of compound **9**.

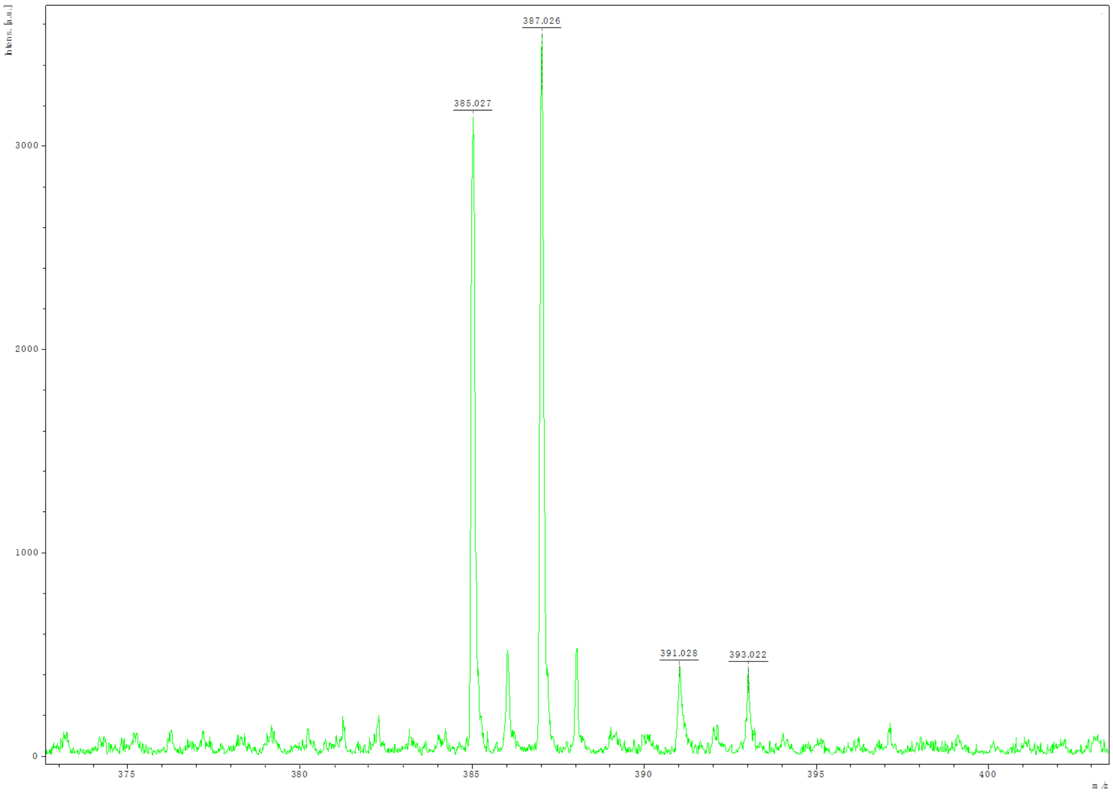

Figure S16. MALDI-TOF-MS spectrum of **9** (*m/z* calculated for $C_{16}H_{17}BrO_6$ $[M+H]^+$: 385.028; Found :385.027).

Phenyl isocyanate (**S4**)

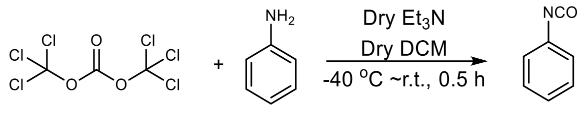

Figure S17. Synthetic route of phenyl isocyanate (**S4**).

Triphosgene (296.7 mg, 1.0 mmol) was dissolved in dry dichloromethane (3.0 mL) in a 25 mL round-bottom flask charged with argon, followed by dropwise addition of aniline (93.1 mg, 0.5 mmol, dissolved in 1.0 mL dry dichloromethane) and dry triethylamine (0.3 mL, 2.1 mmol) at - 40 ℃. The reaction was moved to room temperature and stirred for 30 min. After completion, the mixture was concentrated under reduce pressure to give a crude product **S4** without further purification for next step.

2-((2-oxo-4-(((phenylcarbamoyl)oxy)methyl)-2H-chromen-7-yl)oxy)ethyl 2-bromo-2-methylpropanoate (**7**)

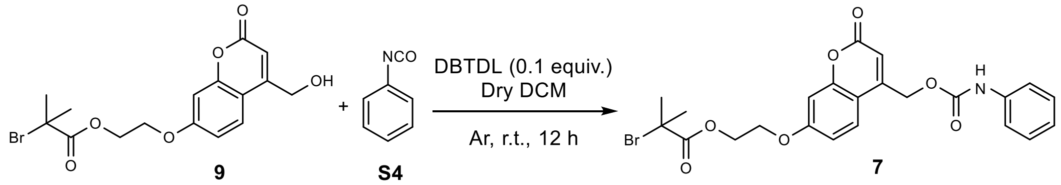

Figure S18. Synthetic route of 2-((2-oxo-4-(((phenylcarbamoyl)oxy)methyl)-2H-chromen-7-yl)oxy)ethyl 2-bromo-2-methylpropanoate (**7**).

2-((4-(hydroxymethyl)-2-oxo-2*H*-chromen-7-yl)oxy)ethyl 2-bromo-2-methylpropanoate **9** (192.6 mg, 0.5 mmol) and dibutyltin dilaurate (34.7 mg, 0.055 mmol) were dissolved in dry dichloromethane (17.0 mL) in a 50 mL round-bottom flask charged with argon, followed by dropwise addition of the solution of above product **S4** in dry dichloromethane (3.0 mL) through syringe with a 450 micron filter. After the mixture was stirred overnight under room temperature, the reaction was quenched by deionized water (10 mL), and extracted with dichloromethane three times (50 mL × 3). The organic layer was combined and dried over anhydrous sodium sulfate, filtrated, evaporated under reduced pressure. The residue was purified by column chromatography (dichloromethane : methanol = 100 : 4) to afford a white solid **7** (246.7 mg, 97.8%). ^{1}H NMR (500 MHz, DMSO-d_6) δ 9.97 (s, 1H), 7.70 (d, J = 9.0 Hz, 1H), 7.49 (d, J = 8.0 Hz, 2H), 7.30 (t, J = 7.8 Hz, 2H), 7.09 (d, J = 2.0 Hz, 1H), 7.02 (t, J = 7.0 Hz, 2H), 6.37 (s, 1H), 5.42 (s, 2H), 4.51 (t, J = 4.0 Hz, 2H), 4.38 (t, J = 4.0 Hz, 2H), 1.89 (s, 6H); ^{13}C NMR (125 MHz, DMSO-d_6) δ 171.2, 161.9, 160.4, 155.3, 153.1, 151.6, 139.2, 129.3, 126.3, 123.2, 118.7, 113.2, 111.0, 109.4, 102.2, 66.8, 64.3, 61.8, 57.4, 30.7. MALDI-TOF-MS *m/z* calculated for $C_{23}H_{22}BrNO_7$ [M]: 503.058; Found: 503.059.

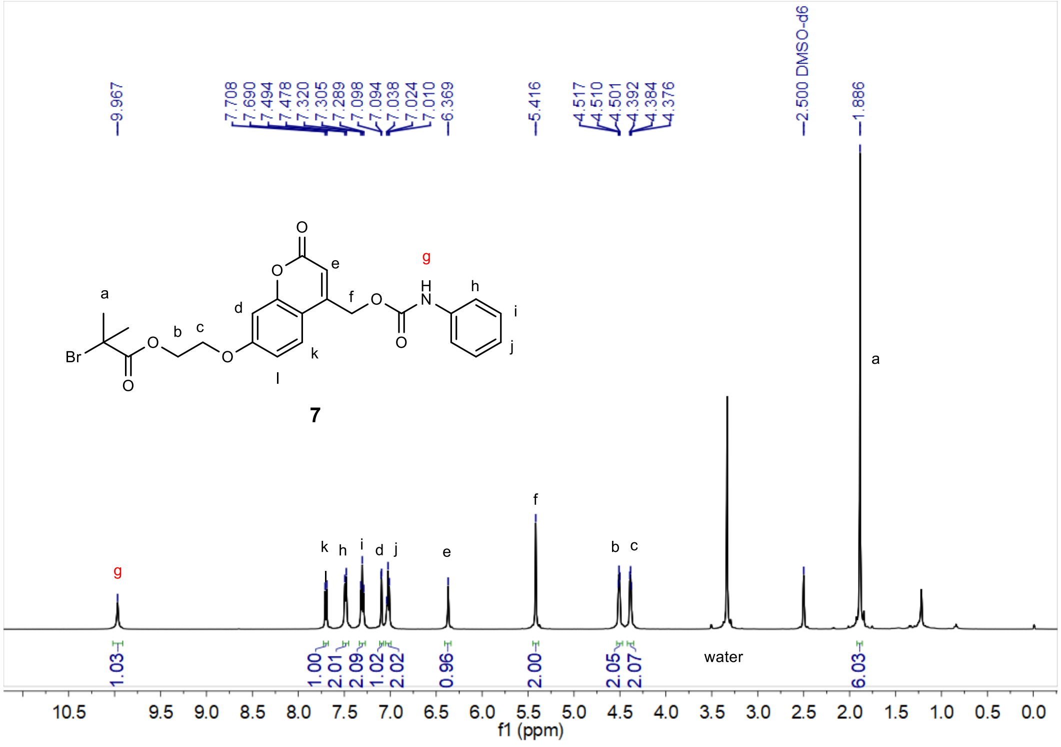

Figure S19. ^{1}H NMR spectrum of compound **7**.

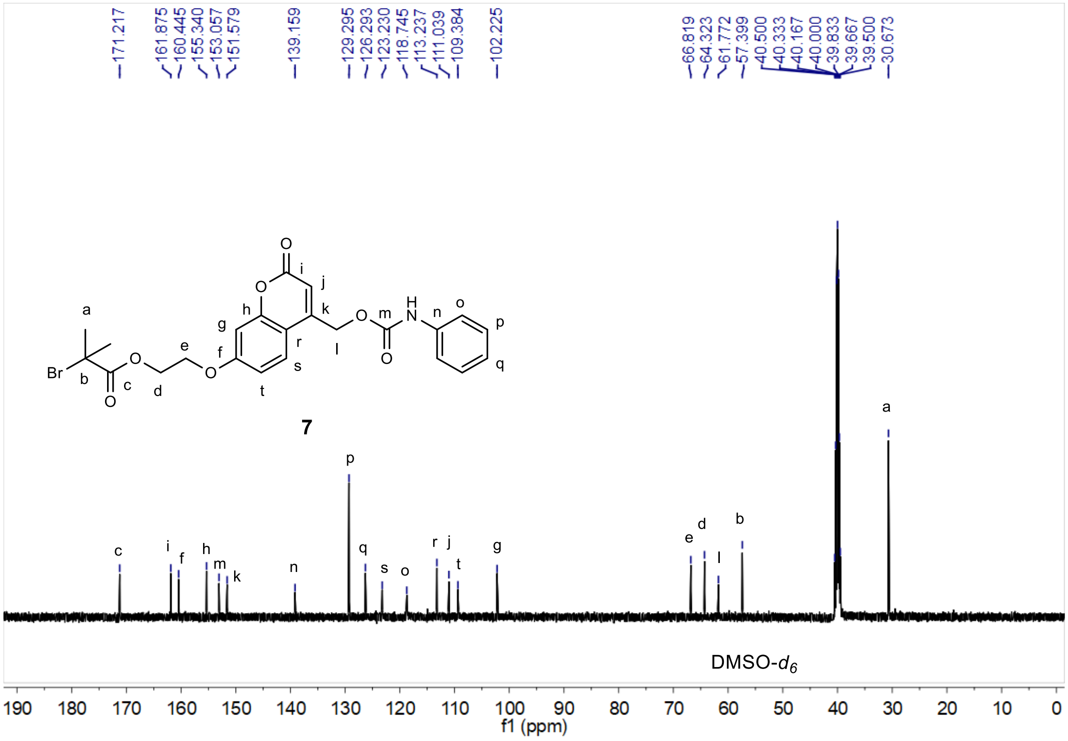

Figure S20. ^{13}C NMR spectrum of compound **7**.

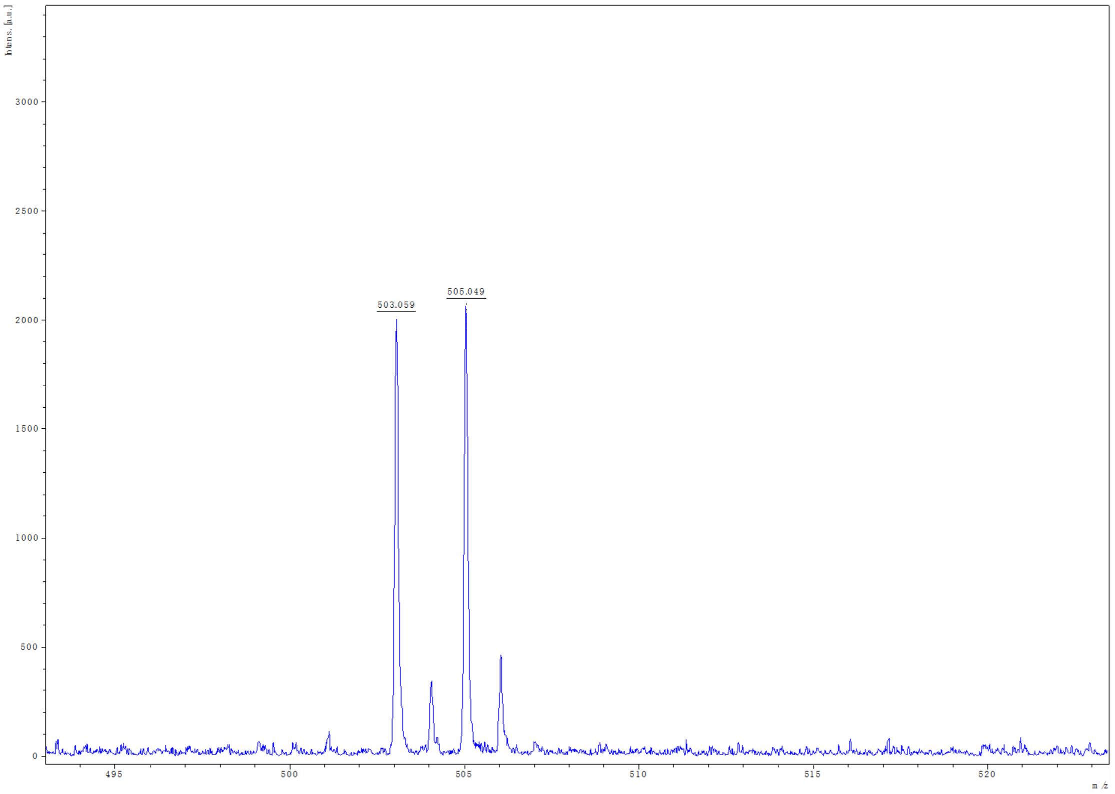

Figure S21. MALDI-TOF-MS spectrum of **7** (*m/z* calculated for $C_{23}H_{22}BrNO_7$ [M]: 503.058; Found :503.059).

(((6a*S*,6b*S*,12b*R*,12c*R*)-6,7-dioxo-12b,12c-bis(((phenylcarbamoyl)oxy)methyl)-6,6a,6b,7,12b,12c-hexahydrocyclobuta[1,2-c:4,3-c']dichromene-3,10-diyl)bis(oxy))bis(ethane-2,1-diyl) bis(2-bromo-2-methylpropanoate) (**8**)

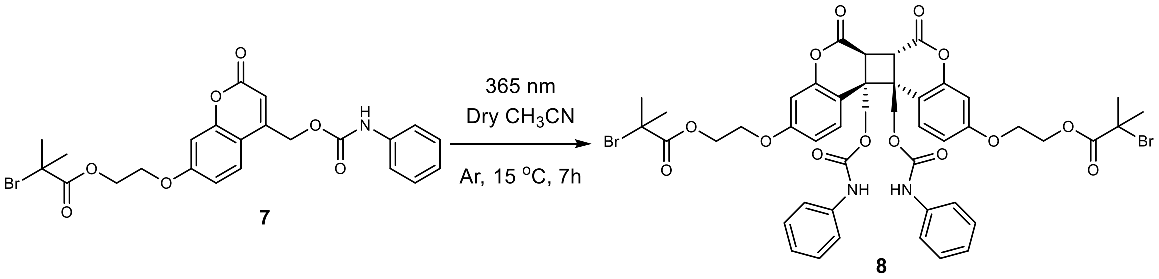

Figure S22. Synthetic route of (((6a*S*,6b*S*,12b*R*,12c*R*)-6,7-dioxo-12b,12c-bis(((phenylcarbamoyl)oxy)methyl)-6,6a,6b,7,12b,12c-hexahydrocyclobuta[1,2-c:4,3-c']dichromene-3,10-diyl)bis(oxy))bis(ethane-2,1-diyl) bis(2-bromo-2-methylpropanoate) (**8**).

The synthesis was adapted from the literature[8]. **7** (151.2 mg, 0.3 mmol) and benzophenone (328.0 mg, 5.0 mmol) were suspended in dry acetonitrile (30 mL) in a 50 mL Schlenk flask charged with argon. Under irradiation of ultraviolet light (45 W, wavelength = 365 nm), the reaction was stirred at 15 ℃ for 7 h. After completion, the mixture was concentrated and the residue was purified by column chromatography (dichloromethane : methanol = 100 : 2) and recrystallization to give a white solid **8** (120.1 mg, 78.9%). ^1H NMR

(500 MHz, $CDCl_3$) δ 7.35-7.30 (m, 8H), 7.09-7.05 (m, 4H), 6.80-6.76 (m, 4H), 6.48 (d, J = 2.5 Hz, 2H), 4.44 (t, J = 4.8 Hz, 4H), 4.33 (d, J = 11.5 Hz, 2H), 4.10-4.05 (m, 2H), 4.02 (d, J = 9.0 Hz, 4H), 3.96-3.92 (m, 2H), 1.93 (s, 12H); ^{13}C NMR (125 MHz, $CDCl_3$) δ 171.5, 160.0, 152.5, 151.3, 137.1, 129.1, 126.1, 123.9, 118.9, 118.9, 112.7, 110.5, 103.6, 65.8, 63.6, 60.4, 55.3, 49.2, 42.1, 30.7. MALDI-TOF-MS *m/z* calculated for $C_{46}H_{44}Br_2N_2O_{14}$ $[M+H]^+$: 1007.123; Found: 1007.134.

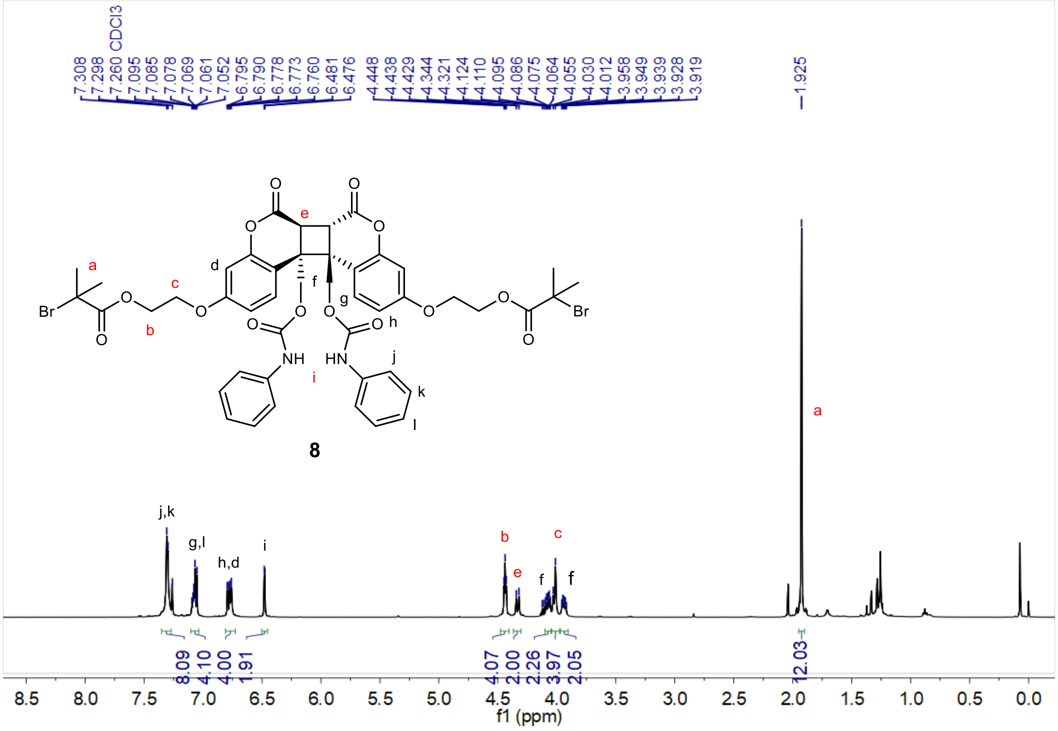

Figure S23. ^{1}H NMR spectrum of compound **8**.

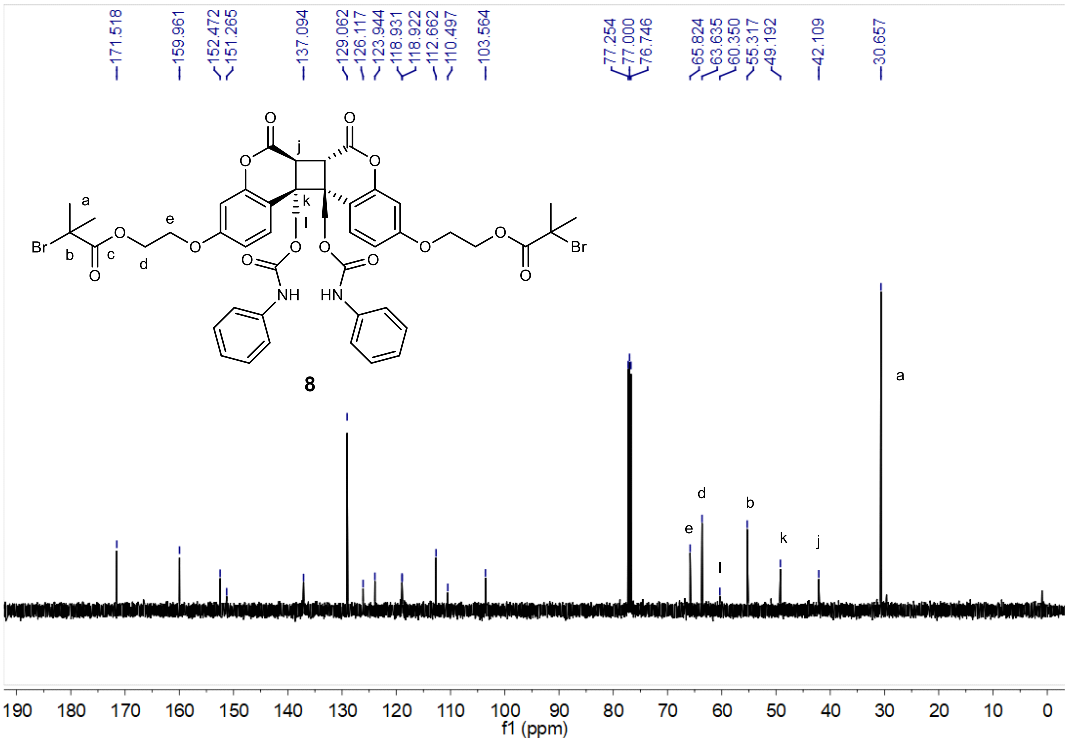

Figure S24. ^{13}C NMR spectrum of compound 8.

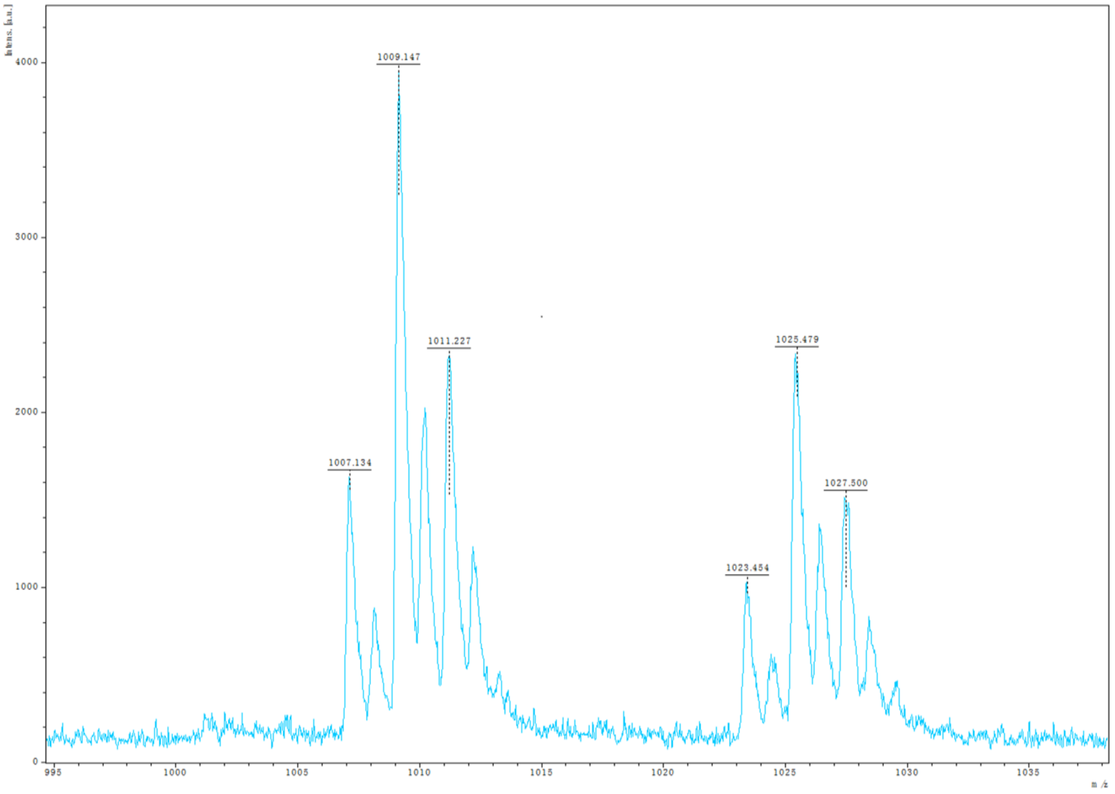

Figure S25. MALDI-TOF-MS spectrum of **8** (*m/z* calculated for $C_{46}H_{44}Br_2N_2O_{14}$ $[M+H]^+$: 1007.123; Found :1007.134).

V. Polymer Synthesis

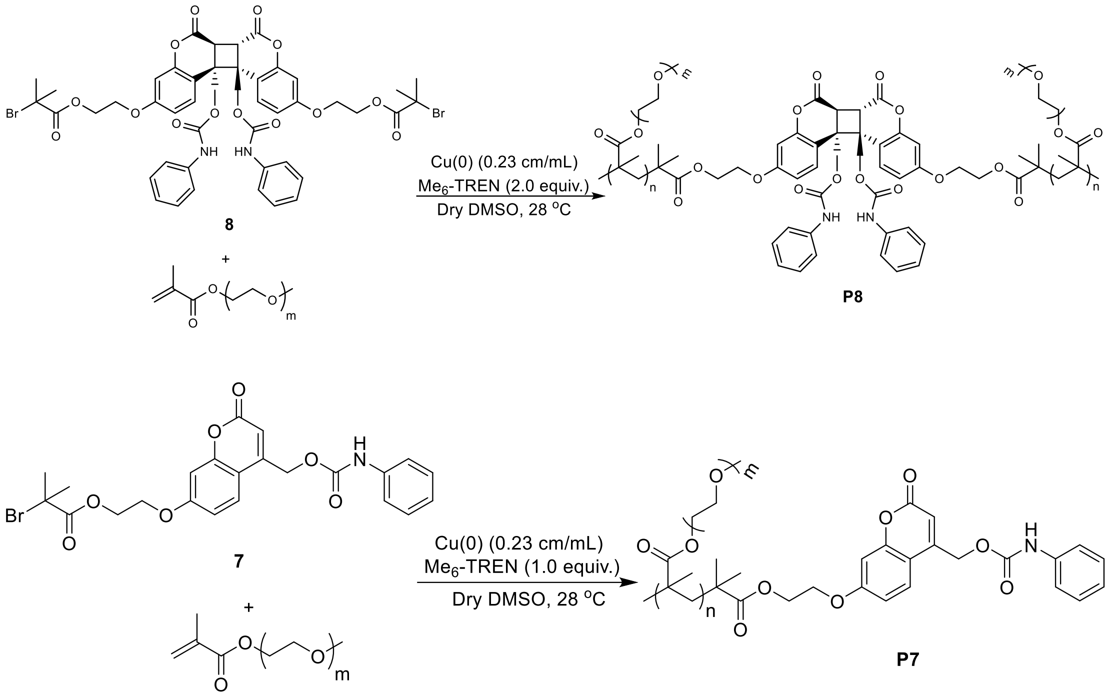

Figure S26. Syntheses of **P8** and **P7**.

Under argon atmosphere, a Schlenk flask with a side arm was charged respectively with initiator **8** (25.2 mg, 0.025 mmol), copper wire (12.1 cm, diameter = 0.64 mm, polished by abrasive paper), Me_6TREN (11.5 mg, 0.05 mmol), oligo(ethylene glycol) methyl ether methacrylate (OEGMA, M_n = 300, 26.3 mL, 90 mmol) and dry DMSO (26.3 mL). The mixture was subjected to three freeze-pump-thaw cycles and then stirred at 28 °C under vacuum for 3.5 h. Afterwards, the reaction was terminated upon exposure to air. The viscous solution was diluted with tetrahydrofuran (5.0 mL) and precipitated in cold ether three times. The obtained product was dried by vacuum pump to afford a colorless polymer **P8**.

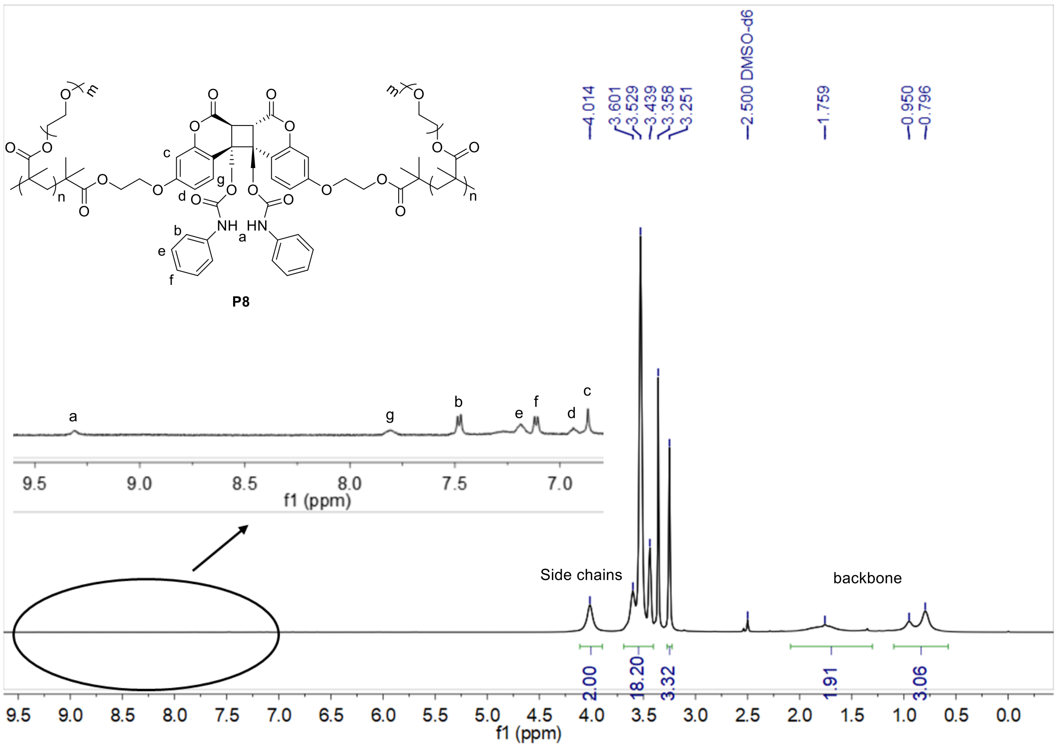

Figure S27. ^{1}H NMR spectrum of **P8** in DMSO-d_6.

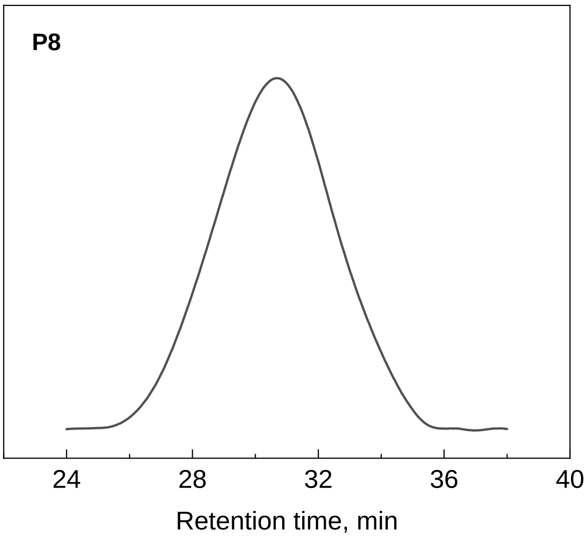

Figure S28. RI output of GPC of **P8** (M_n = 181.5 kDa, $Đ_M$ = 1.72 based on calibration with PMMA standards). The eluant is 5.0 mM NH_4BF_4 in DMF.

The step is similar to the synthesis of polymer **P8**. Under argon atmosphere, a Schlenk flask with a side arm was charged respectively with initiator **7** (25.2 mg, 0.05 mmol), copper wire (12.1 cm, diameter = 0.64 mm, polished by abrasive paper), Me_6TREN (11.5 mg, 0.05 mmol),

oligo(ethylene glycol) methyl ether methacrylate (OEGMA, M_n = 300, 26.3 mL, 90 mmol) and dry DMSO (26.3 mL). The mixture was subjected to three freeze-pump-thaw cycles and then stirred at 28 °C under vacuum for 3.5 h. Afterwards, the reaction was terminated upon exposure to air. The viscous solution was diluted with tetrahydrofuran (5.0 mL) and precipitated in cold ether three times. The obtained product was dried by vacuum pump to afford a colorless polymer **P7**.

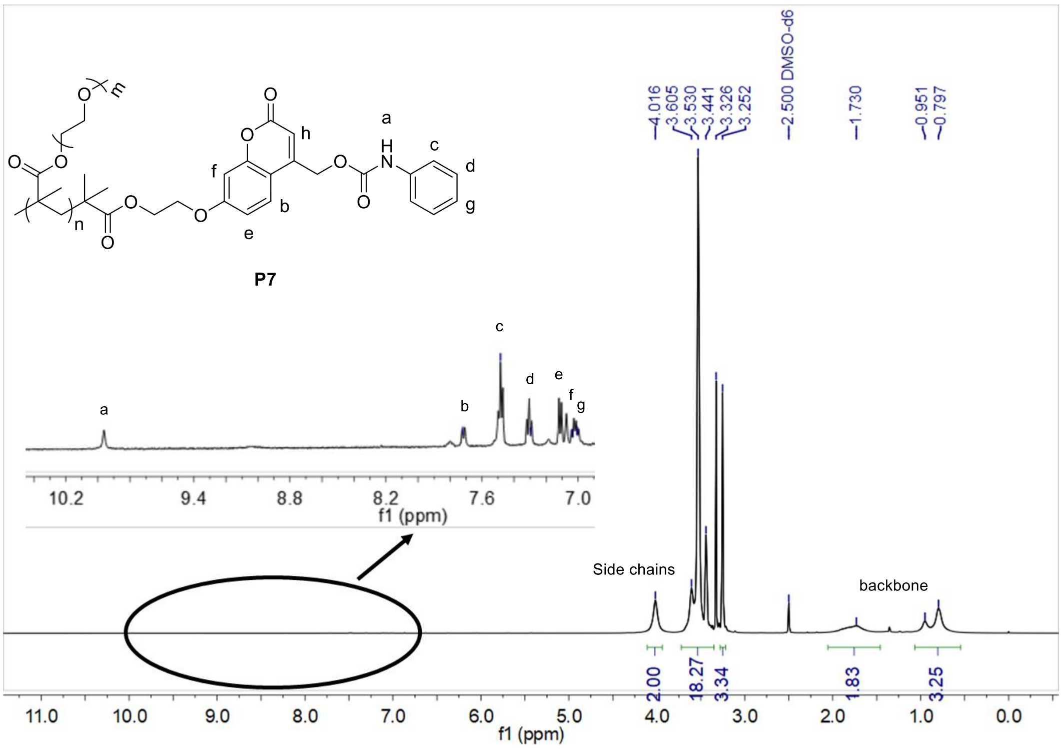

Figure S29. ^{1}H NMR spectrum of **P7**.

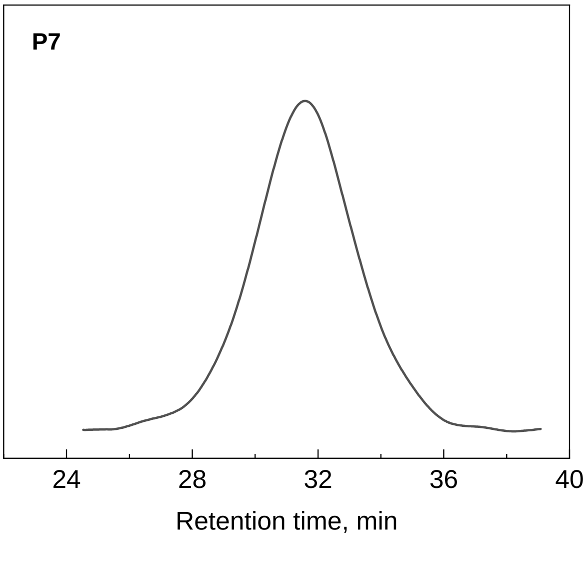

Figure S30. The RI output of GPC of **P7** (M_n = 136.2 kDa, $Đ_M$ = 1.54) in 5.0 mM NH_4BF_4 in DMF.

Masses are based on calibration with PMMA standards.

VI. Physical measurements, material characterization and data processing

1. Estimation of Mechanochemical Activation Yield

Fragmentation of P8 by retro [2 +2] cycloaddition of the coumarin dimer during ultrasound sonication can be represented as:

$$\mathbf{P8} \overset{k}{\rightarrow} 2\,\mathbf{P7}$$

$$a = \frac{dI_{dimer}}{d[dimer]}$$

$$b = \frac{dI_{coumarin}}{d[coumarin]}$$

$$[dimer]_t + \frac{[coumarin]_t}{2} = \frac{I_0}{a}$$

$$y = \frac{\frac{I_t}{I_0} - 1}{\frac{2b}{a} - 1} \quad (eq.S2)$$

where y is the fraction of the dimer that dissociated mechanochemically at sonication time t. a is the slope of the linear dependence of fluorescence intensity of **P8** solution on the molar concentration of the dimer. b the linear dependence of fluorescence intensity of **P7** solution on the molar concentration of the coumarin. $[dimer]_t$ is the concentration of the coumarin dimer at sonication time t. $[coumarin]_t$ is the concentration of the coumarin at sonication time t. I_0 is the fluorescent intensity of the **P8** solution prior to sonication, $I_{(t)}$ is the fluorescent intensity of sonicated **P8** solution at time t.

2. Photochemical release of aniline.

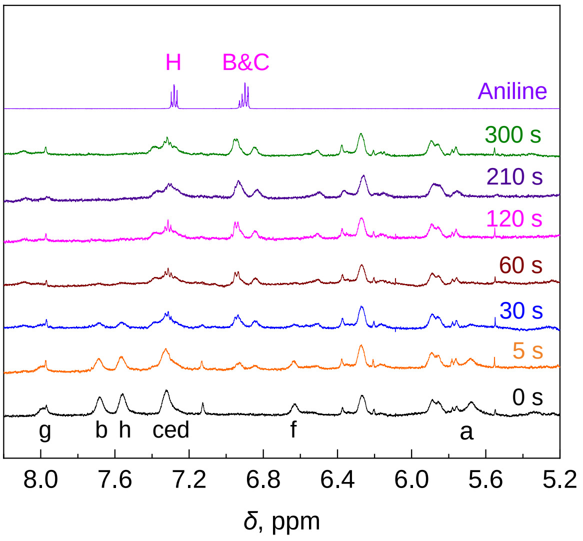

Figure S31. The ^{1}H NMR spectra (500 MHz) of **P7** (a 200 mg/mL, ~ 1.46 mM solution in D_2O) at different irradiation (λ = 365 nm) times and of a 10 mM solution of aniline in D_2O (top trace).

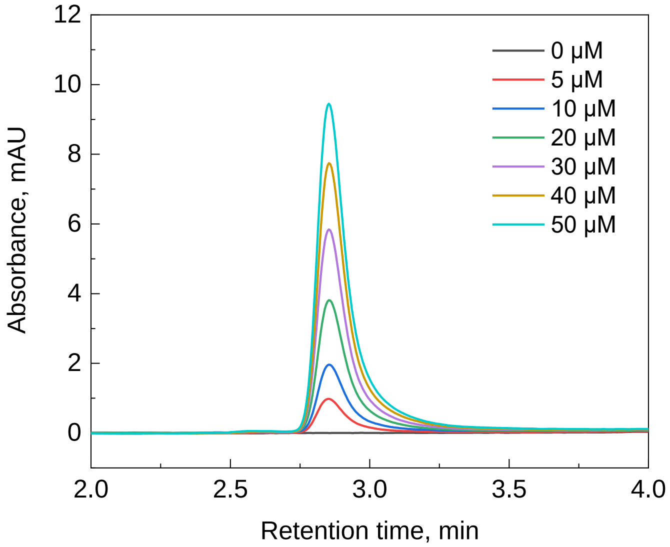

Figure S32. The 280 nm UV-vis detector output of HPLC of a solution of aniline in deionized water at different concentrations.

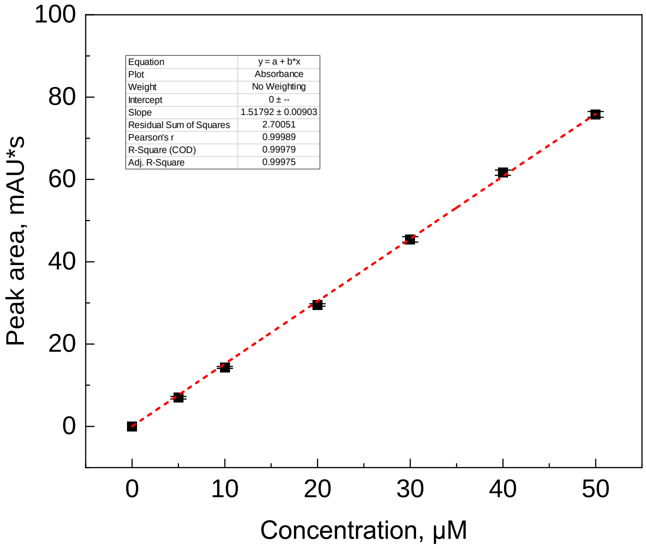

Figure S33. The calibration plot of the HPLC peak area of aniline (absorbance at 280 nm) as a function of its concentration.

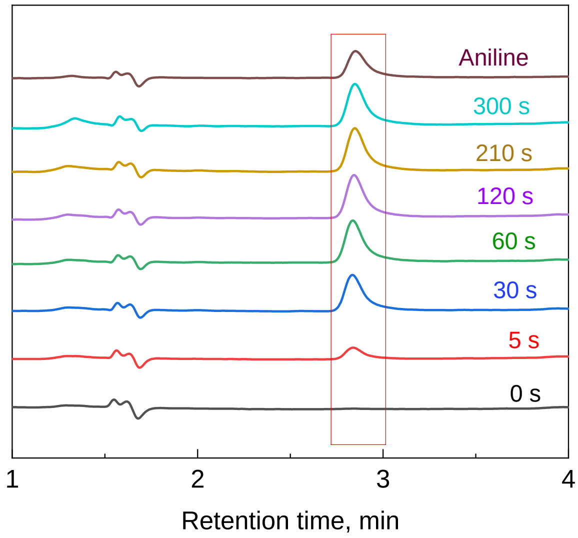

Figure S34. The 280-nm output of the UV-vis detector of HPLC of a 6.0 mg/mL (0.044 mM) solution of **P7** in deionized water at different irradiation (λ = 365 nm) times.

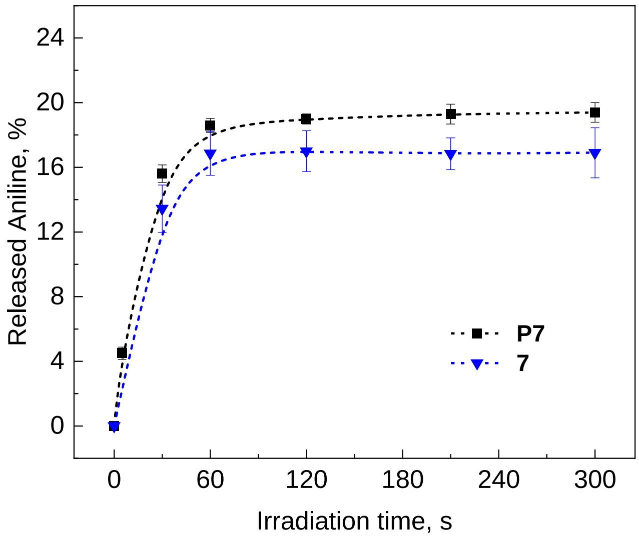

Figure S35. Illustrative examples of the fractions of photochemically released aniline by **7** (as a 10 μM solution in 50% aqueous CH_3OH) and **P7** (as a 6.0 mg/mL, 0.044 mM solution in deionized water) as a function of irradiation time at 365 nm.

The progress of photochemical reaction upon 365 nm irradiation of coumarin-terminated **P7** and of the small-molecule analog **7** was also followed by UV-Vis spectroscopy (Figure S36, Figure S37, Figure S38 and Figure S39), demonstrating a gradual increase in the absorption intensity at 280 nm (aniline) and concomitant decrease in absorbance at 322 nm (coumarin/aniline adduct).

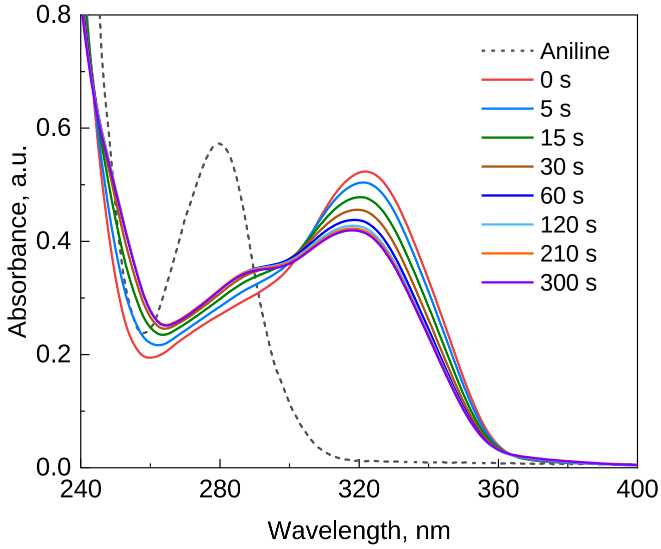

Figure S36. UV/vis spectra of **P7** (6.0 mg/mL, 0.044 mM) in H_2O at different irradiation (λ = 365

nm) times; the spectrum of aniline at 0.4 mM is shown for reference.

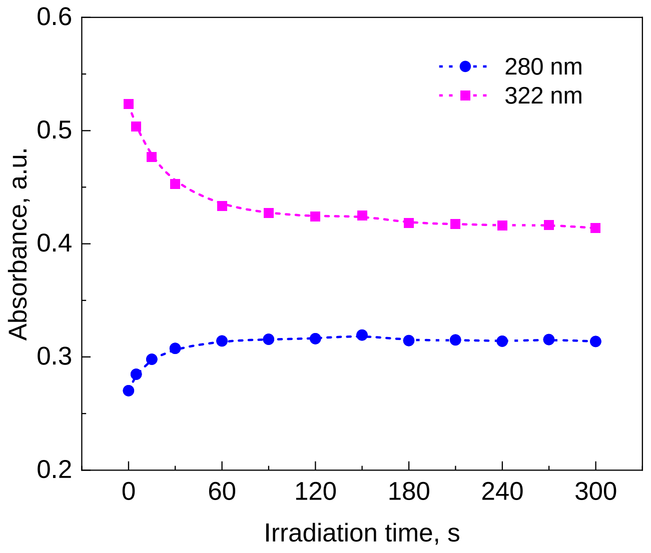

Figure S37. The absorbance at 280 nm and 322 nm of **P7** (6.0 mg/mL, 0.044 mM in H_2O) at different irradiation (λ = 365 nm) times.

The photochemistry of **7** was also investigated by UV-Vis and the results are shown in Figure S38 and Figure S39, and the phenomenon was similar to that of **P7**.

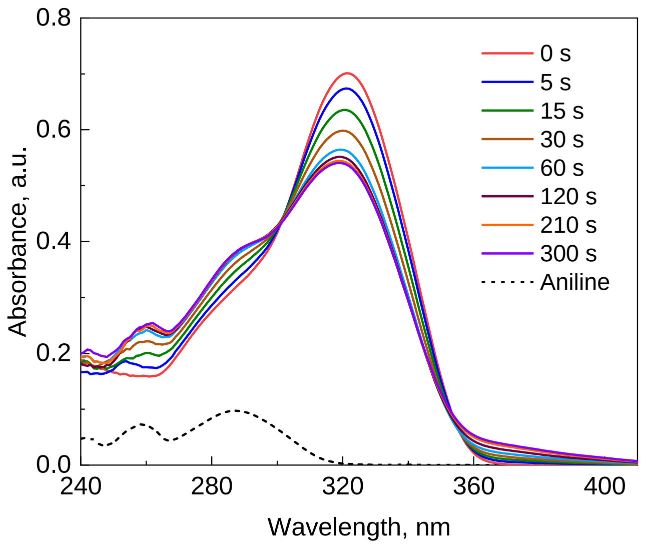

Figure S38. UV/vis spectra of **7** (as a 0.05 mM solution in DMF/H_2O at 2:1 by volume) at different irradiation (λ = 365 nm) times; the spectrum of aniline at 0.05 mM is shown for reference.

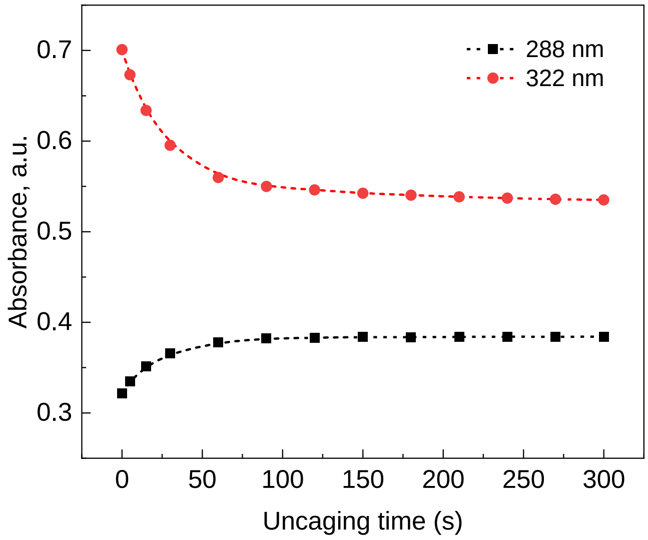

Figure S39. The absorbance at 288 nm and 322 nm of **7** (as a 0.05 mM solution in DMF/H_2O (2:1 by volume) at different irradiation (λ = 365 nm) times.

We confirmed dimerization of coumarin under 365 nm UV irradiation by analyzing by HPLC a solution of **9** and by GPC a solution of **P7** irradiated at 365 nm ((Figure S40-Figure S41). In HPLC samples the intensity of the peak at 7.5 min (**9**) gradually decreased and a new peak appeared at 5.8 min, which we attribute to dimer $\mathbf{9}_2$. GPCs of the irradiated solution of **P7** developed a shoulder at higher molecular mass, with M_n of the sample increasing from 136.6 kDa to 161.3 kDa after 5 min of irradiation, suggesting photodimerization of coumarin-terminated **P7** to **P8**.

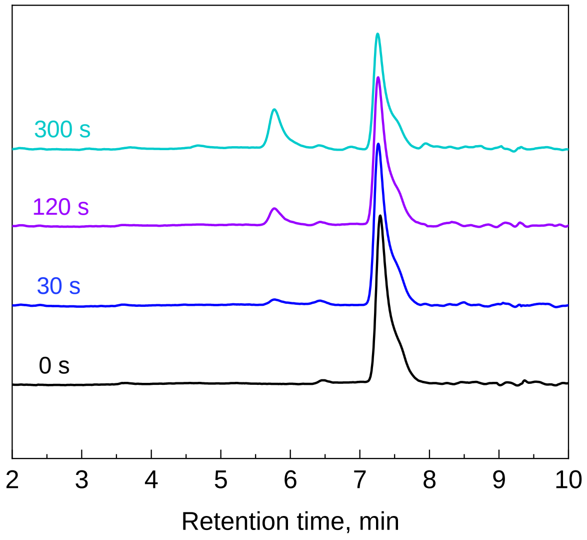

Figure S40. RI detector output of HPLC of 10 μM of **9** in 50% aqueous CH_3OH at different irradiation (λ = 365 nm) times. A peak at 5.8 min is the dimer.

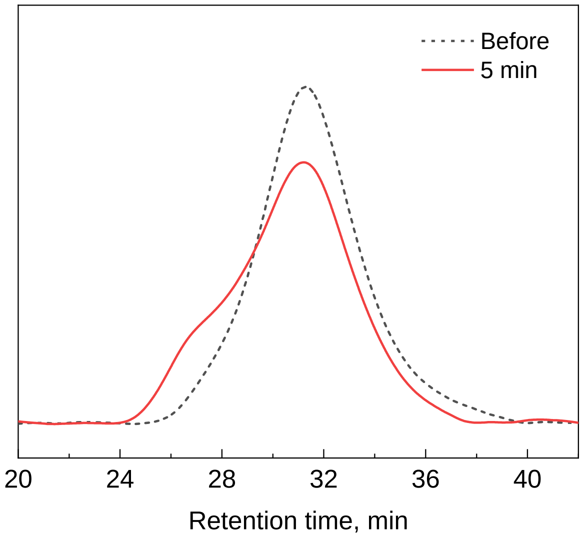

Figure S41. RI detector output of GPC of **P7** before (black dashed line, M_n = 136.6 kDa, $Đ_M$ = 1.80) and after (red solid line, M_n = 161.3 kDa, $Đ_M$ = 2.60) irradiation with 365 nm UV light for 5 min.

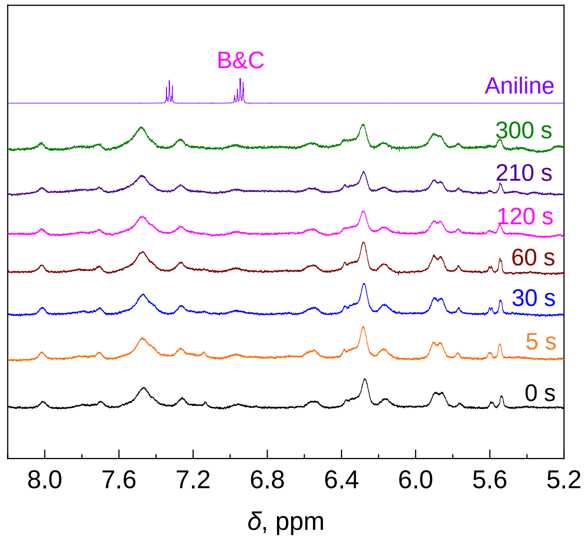

Figure S42. ^{1}H NMR spectra of **P8** (200 mg/mL, ~1 mM in D_2O) at different irradiation (λ = 365 nm) times; for reference, the spectrum of a 10 mM solution of aniline in D_2O is shown as the top trace.

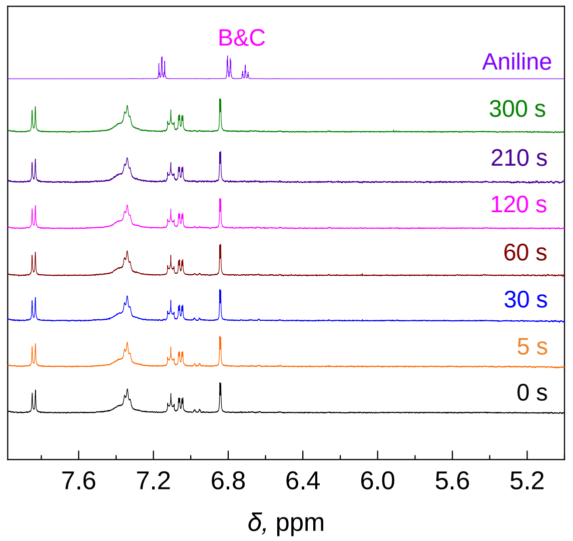

Figure S43. ^{1}H NMR spectra of **8** as a 0.4 mM in DMF-d_7/D_2O (2:1 ratio by volume) at different irradiation (λ = 365 nm) times; for reference, the spectrum of a 1.5 mM solution of aniline in the same solvent is shown at the top.

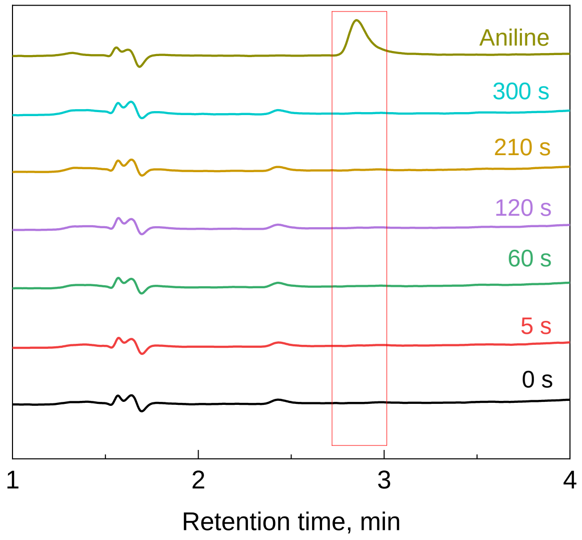

Figure S44. Absorbance at 280 nm in HPLC of **P8** (6.0 mg/mL, 0.033 mM) in deionized water at different irradiation (λ = 365 nm) times.

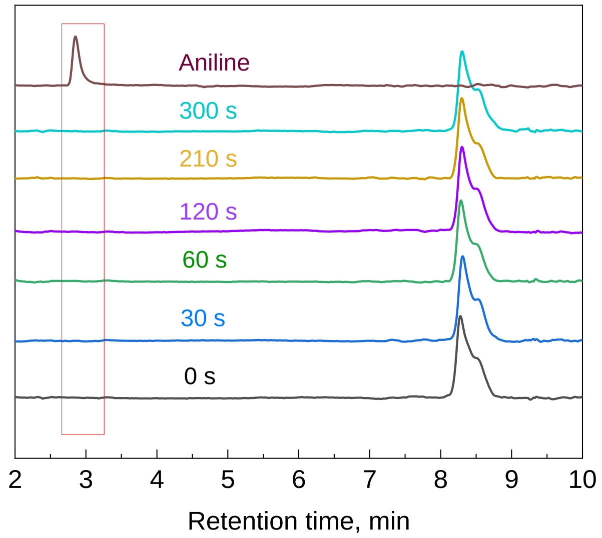

Figure S45. Absorbances at 280 nm of HPLC of **8** (as a 15 µM in 50% aqueous CH_3OH) at different irradiation (λ = 365 nm) times; for reference, the HPLC of a 5 µM solution of aniline in deionized water is shown at the top.

Absorption spectra of irradiated solutions of **P8** and **8** did not change under irradiation at 365 nm (Figure S46 and Figure S47), indicating no photochemical reactions, such as aniline release or dimer photodissociation.

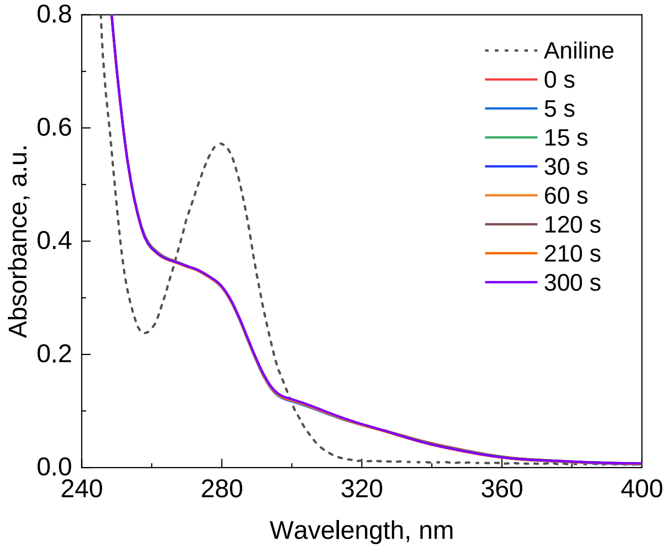

Figure S46. UV-Vis spectra of **P8** (6.0 mg/mL, 0.033 mM) in H_2O at different irradiation (λ = 365 nm) times and of a 0.4 mM solution of aniline in H_2O.

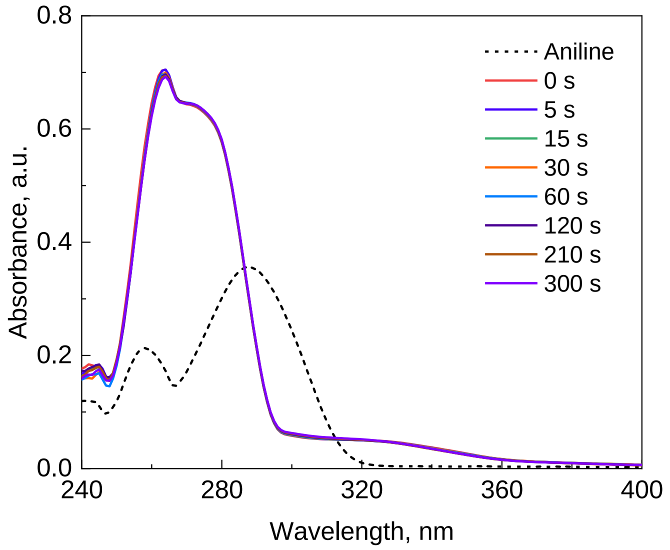

Figure S47. UV/vis spectra of a 0.10 mM solution of **8** in DMF/H_2O (2:1 by volume) at different irradiation (λ = 365 nm) times and of 0.20 mM solution of aniline in the same solvent.

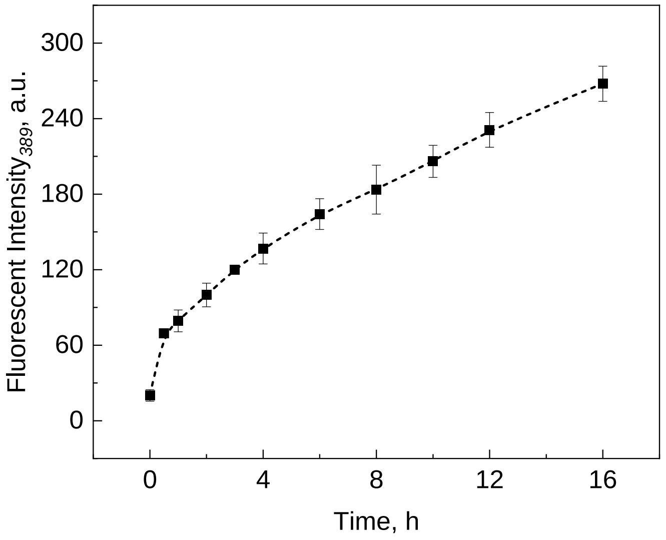

Figure S48. The fluorescent intensity at 389 nm of a solution of **P8** (6.0 mg/mL, 0.033 mM in DI water) as a function of sonication times (λ_{ex}= 322 nm).

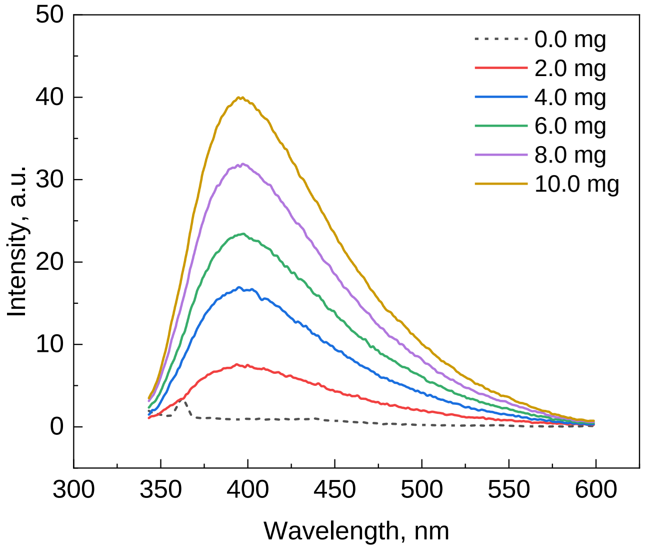

Figure S49. Fluorescence emission spectra of aqueous solutions of **P8** at different concentrations (λ_{ex} = 322 nm).

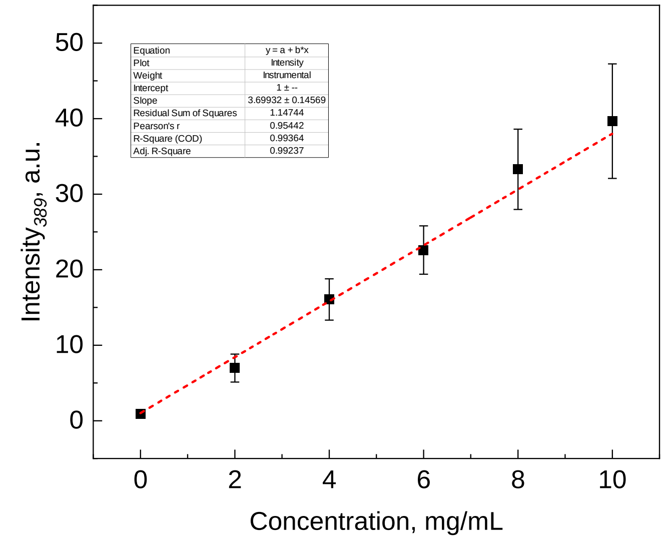

Figure S50. The fluorescence emission intensity at 389 nm of an aqueous solution **P8** as a function of its concentration. (λ_{ex} = 322 nm).

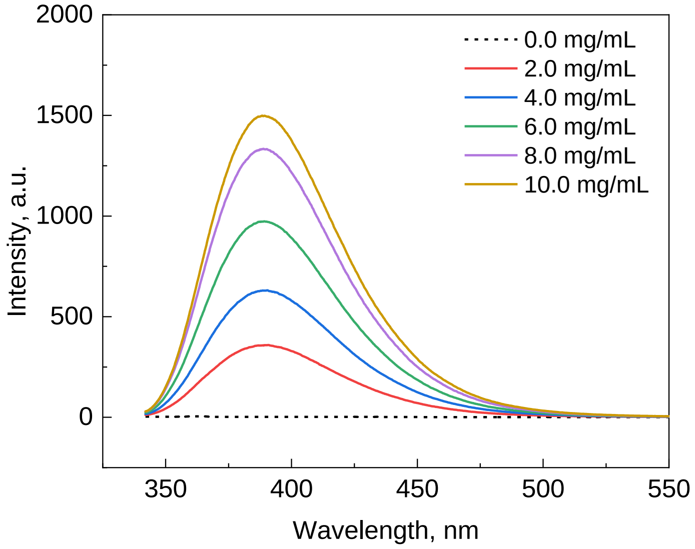

Figure S51. The fluorescence emission spectra of an aqueous solution of **P7** at different concentrations (λ_{ex} = 322 nm).

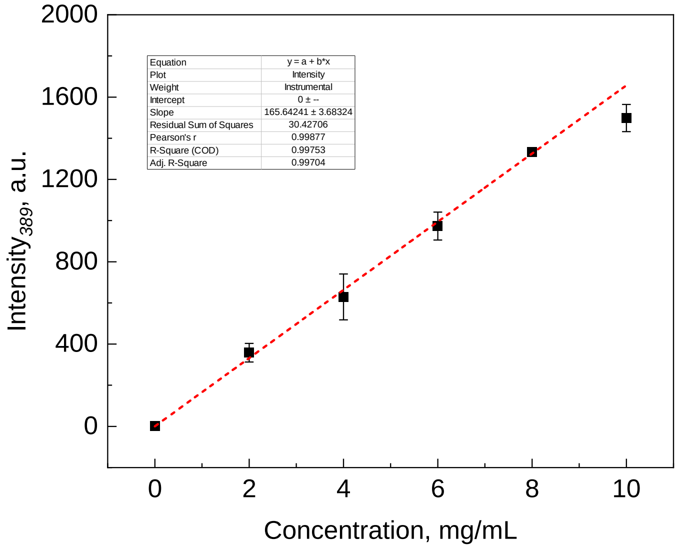

Figure S52. The fluorescence emission intensity at 389 nm of aqueous solutions **P7** as a function of its concentration (λ_{ex} = 322 nm).

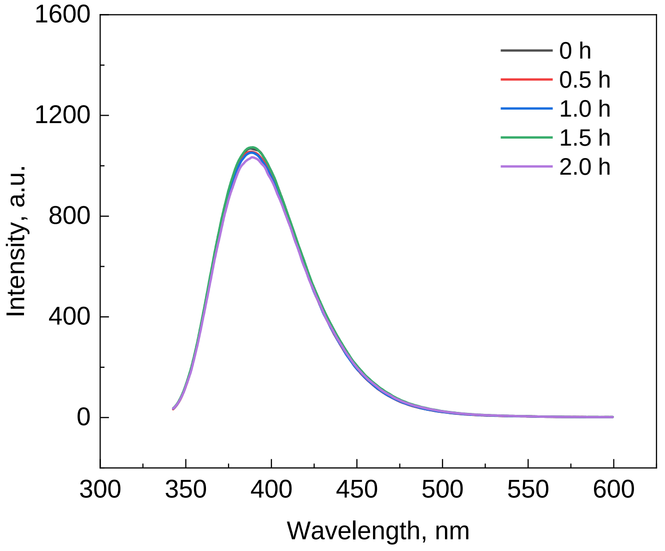

Figure S53. Fluorescent spectra of of a solution **P7** (6.0 mg/mL, 0.044 mM in DI water) at different sonication times (λ_{ex} = 322 nm).

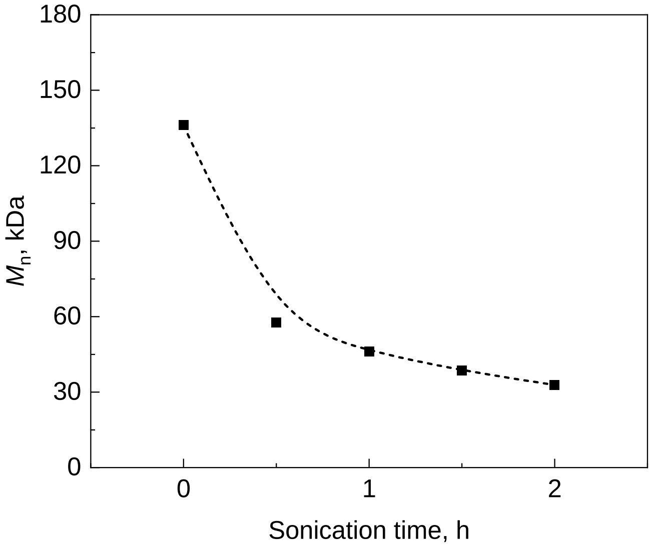

Figure S54. The number-average molar mass, M_n, of a sonicated solution of **P7** (0.9 mg/mL in DMF containing 5 mM NH_4BF_4) at different sonication times. For each time, a 300 μL aliquote of this solution was freeze-dried and then dissolved in DMF containing 5 mM NH_4BF_4 for GPC analysis.

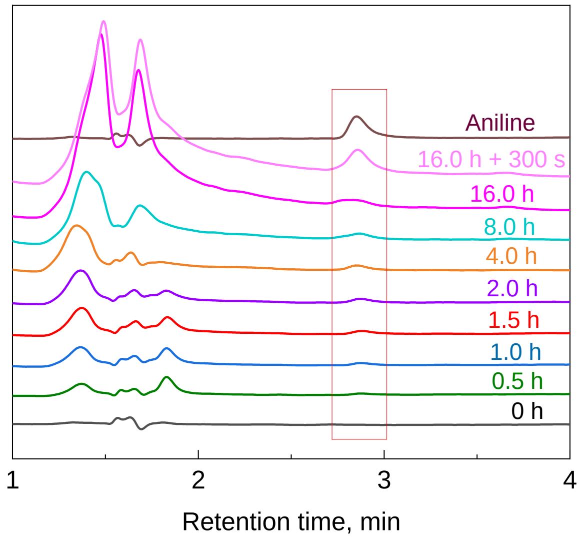

Figure S55. Absorption at 280 nm of HPLC of a solution of **P7** (6.0 mg/mL, 0.044 mM in DI water) sonicated for 0 h, 0.5 h, 1.0 h, 1.5 h, 2.0 h, 4.0 h, 8.0 h, 16.0 h; sonicated for 16 h and then irradiated at 365 nm UV light for 300 s, and of a 5 μM solution of aniline in DI water.

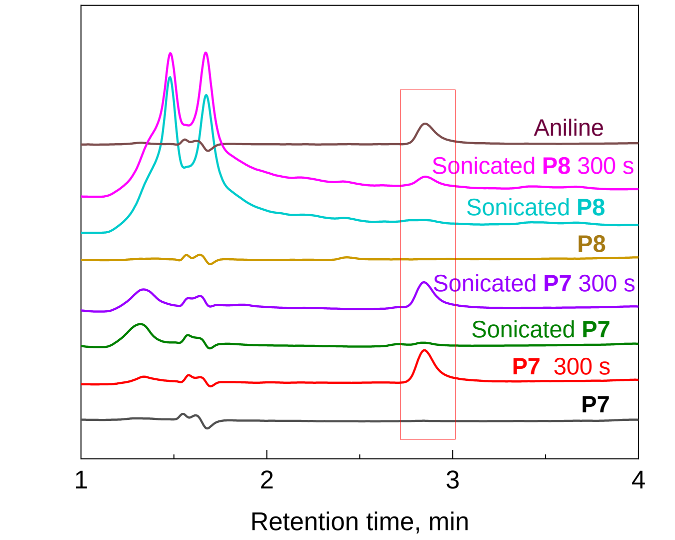

Figure S56. Absorption at 280 nm of HPLC of aqueous solutions of **P7** (6.0 mg/mL, 0.044 mM): before sonication or irradiation, after irradiation at 365 nm for 300 s; after sonication for 2 h; and after 2 h-sonication followed by irradiation at 365 nm for 300 s; of aqueous solutions of **P8** (6.0 mg/mL, 0.033 mM): before sonication or irradiation, after sonication for 16 h; after sonication for 16 h and irradiation at 365 nm for 300 s; and of a 5 μM aqueous solution of aniline.

Absorption spectra of an aqueous solution of **P8** sonicated for 16 h followed by irradiation at 365 nm displayed a gradually decreasing absorbance at 322 nm, corresponding to the coumarin/aniline construct (Figure S57).

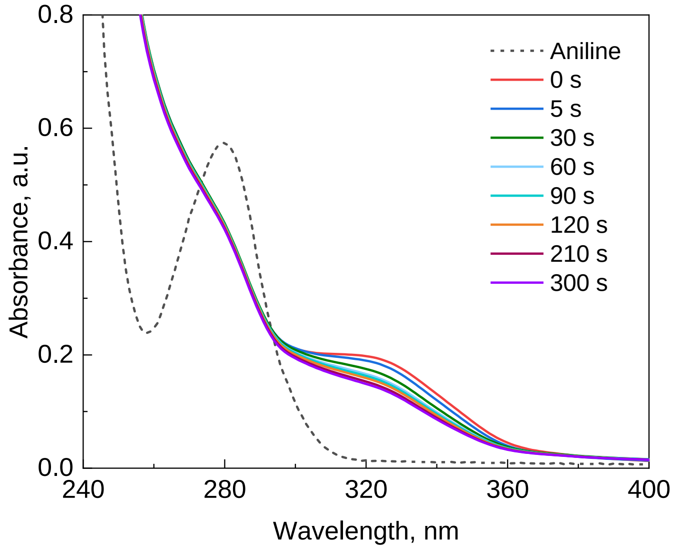

Figure S57. UV-Vis spectra of a solution of **P8** (6.0 mg/mL, 0.033 mM in DI water) sonicated for

16 h and irradiated at 365 nm for the time shown; for reference, the spectrum of a 0.4 mM solution of aniline in DI water is shown. Absorption of **P8** at <300 nm is dominated by the backbone with some contribution from the coumarin dimer.

3. Photomechanically controlled gelation.

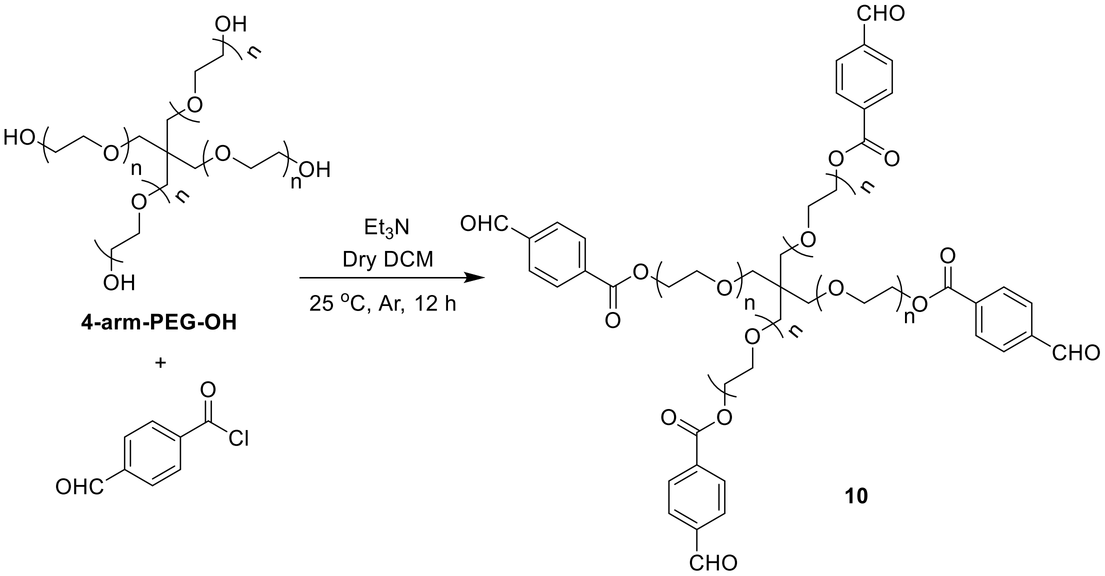

Figure S58. Synthesis of polymer benzaldehyde terminating 4-arm polyethylene glycol **10**.

Hydroxy-terminated 4-arm star PEG (M_n = 10 kDa, $Đ_M$ = 1.02, 10.8376 g, 1.083 mmol) and triethylamine (0.6580 g, 6.5 mmol) were dissolved in dry dichloromethane (80.0 mL) in a 250 mL round-bottom flask charged with argon, followed by dropwise addition of a solution of 4-formly benzoyl chloride (1.0962 g, 6.5 mmol) in dry dichloromethane (20.0 mL) through syringe at 0 °C. The resulting mixture was moved to room temperature and stirred for 16 h. Afterwards, the reaction was quenched by saturated sodium bicarbonate (40 mL) at room temperature for 0.5 h and extracted with dichloromethane five times (100 mL × 3). The organic solvent was combined and washed with saturated brine, dried over anhydrous sodium sulfate, filtered, and evaporated in vacuo. The residue was precipitated from dichloromethane with cold ether three times to afford **10** as a white powder (11.0311 g, 96.7% yield, 74.0% modification). The modification rate was determined by comparing the ^{1}H NMR peak of the benzaldehyde group on **10** with the CH_2 peak of ethylene glycol (EG) repeat unit (3.5 ppm, Fig. S59). Each molecule of the precursor has 4x227=908 such protons, the measured 2.96:909 ratio of intensities of the two peaks suggests ~75% of the terminal OH groups in the precursor were terminated with benzaldehyde. ^{1}H NMR (500 MHz, DMSO-d_6) δ 10.12 (s, 1H), 8.16 (d, J = 8.0 Hz, 2H), 8.06 (d, J = 8.5 Hz, 2H), 4.44-3.76 (m, 909H).

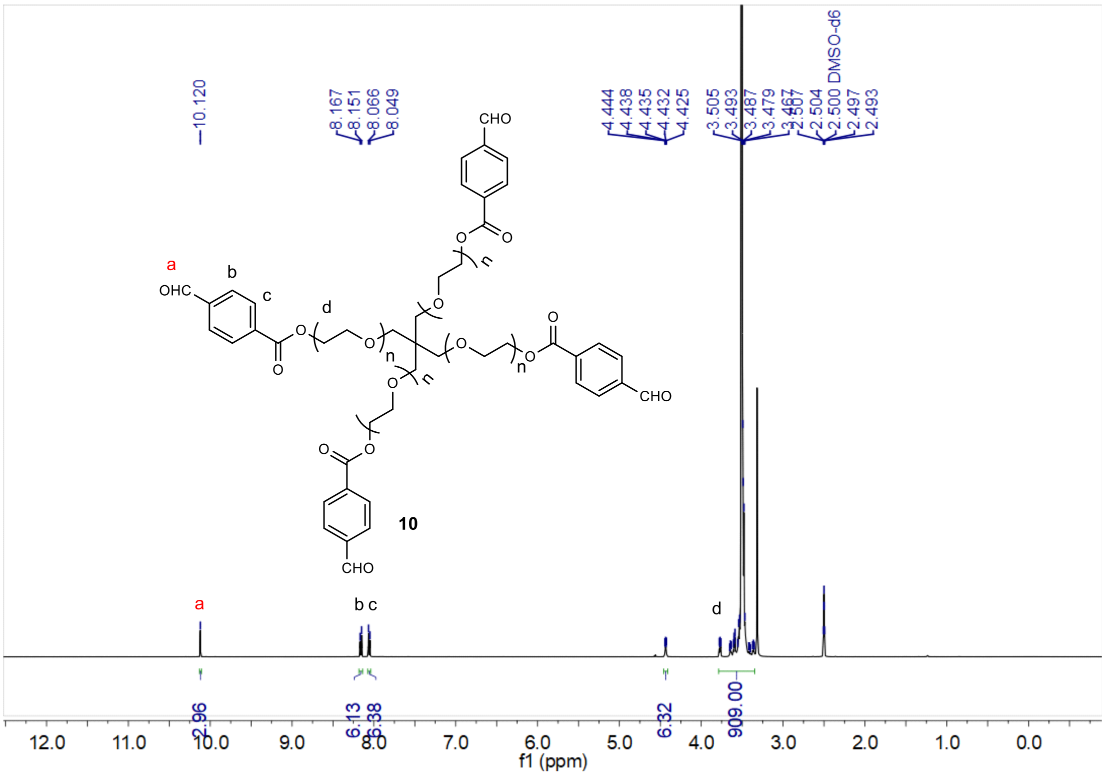

Figure S59. ^{1}H NMR spectrum of **10**.

Table S3. Summary of the gelation of samples resulting from different additive.

Sample	D	E	F	G
polymer	**P8**	none	none	**P7**
Sonication time, h	16	0	0	0
Irradiation time, min	0	0	0	0
Additive aniline, mg	0.16	0.16	0	0
210 min after mixing	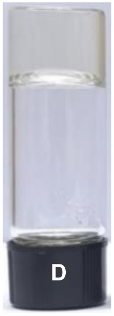	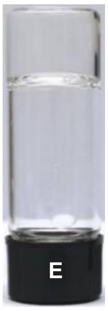	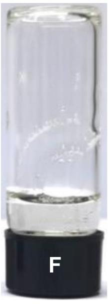	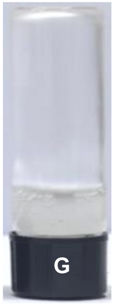

Samples for gelation experiments were prepared by dissolving 100.0 mg of freeze-dried polymer **P7** or **P8** (whether as-synthesized or sonicated) in 900 μL 100 mM phosphate buffer solution (pH = 7.2). For samples A and C, the solutions were then irradiated for 5 min with a

365 nm diode. To either irradiated (samples A and C) or as-prepared (samples B, D-G) solution of **10** (300 mg, Figure S58-S59) 100 μL of 100 mM phosphate buffer containing adipic dihydrazide (at 104.0 mg/mL concentration) were added. The results verify that the gelation is accelerated by the aniline released from mechanochemically activated **P8** upon UV irradiation.

VII. References

1. Akbulatov, S.; Tian, Y.; Huang, Z.; Kucharski, T. J.; Yang, Q.-Z.; Boulatov, R., Experimentally realized mechanochemistry distinct from force-accelerated scission of loaded bonds. *Science* **2017,** *357* (6348), 299-303.
2. Hermes, M.; Boulatov, R., The Entropic and Enthalpic Contributions to Force-Dependent Dissociation Kinetics of the Pyrophosphate Bond. *J. Am. Chem. Soc.* **2011,** *133* (Copyright (C) 2012 American Chemical Society (ACS). All Rights Reserved.), 20044-20047.
3. Cramer, C. J., *Essentials of Computational Chemistry*. 2nd ed. ed.; Wiley: Chichester, 2004.
4. Kucharski, T. J.; Boulatov, R., The physical chemistry of mechanoresponsive polymers. *J. Mater. Chem.* **2011,** *21* (Copyright (C) 2012 American Chemical Society (ACS). All Rights Reserved.), 8237-8255.
5. Ochterski, J. W. Vibrational Analysis in Gaussian. http://gaussian.com/vib/ (accessed March 3).
6. Shaughnessy, K. H.; Kim, P.; Hartwig, J. F., A fluorescence-based assay for high-throughput screening of coupling reactions. Application to Heck chemistry. *J. Am. Chem. Soc.* **1999,** *121* (10), 2123-2132.
7. Brimioulle, R.; Guo, H.; Bach, T., Enantioselective intramolecular [2+2] photocycloaddition reactions of 4-substituted coumarins catalyzed by a chiral Lewis acid. *Chemistry-A European Journal* **2012,** *18* (24), 7552-60.
8. Yu, X.; Scheller, D.; Rademacher, O.; Wolff, T., Selectivity in the photodimerization of 6-alkylcoumarins. *J. Org. Chem.* **2003,** *68* (19), 7386-7399.
